# Dose and timing effects of caffeine on subsequent sleep: a randomized clinical crossover trial

**DOI:** 10.1093/sleep/zsae230

**Published:** 2024-10-08

**Authors:** Carissa L Gardiner, Jonathon Weakley, Louise M Burke, Francesca Fernandez, Rich D Johnston, Josh Leota, Suzanna Russell, Gabriella Munteanu, Andrew Townshend, Shona L Halson

**Affiliations:** School of Behavioural and Health Sciences, Australian Catholic University, Brisbane, Australia; Sports Performance, Recovery, Injury and New Technologies (SPRINT) Research Centre, Australian Catholic University, Brisbane, Queensland, Australia; School of Behavioural and Health Sciences, Australian Catholic University, Brisbane, Australia; Sports Performance, Recovery, Injury and New Technologies (SPRINT) Research Centre, Australian Catholic University, Brisbane, Queensland, Australia; Carnegie Applied Rugby Research (CARR) Centre, Institute of Sport, Physical Activity and Leisure, Leeds Beckett University, Leeds, UK; Exercise and Nutrition Research Program, Mary MacKillop Institute for Health Research, Australian Catholic University, Melbourne, Victoria, Australia; School of Behavioural and Health Sciences, Australian Catholic University, Brisbane, Australia; School of Behavioural and Health Sciences, Australian Catholic University, Brisbane, Australia; Sports Performance, Recovery, Injury and New Technologies (SPRINT) Research Centre, Australian Catholic University, Brisbane, Queensland, Australia; Carnegie Applied Rugby Research (CARR) Centre, Institute of Sport, Physical Activity and Leisure, Leeds Beckett University, Leeds, UK; School of Psychological Sciences, Monash University, Melbourne, Victoria, Australia; Turner Institute for Brain and Mental Health, Monash University, Melbourne, Victoria, Australia; School of Behavioural and Health Sciences, Australian Catholic University, Brisbane, Australia; Sports Performance, Recovery, Injury and New Technologies (SPRINT) Research Centre, Australian Catholic University, Brisbane, Queensland, Australia; School of Behavioural and Health Sciences, Australian Catholic University, Brisbane, Australia; Sports Performance, Recovery, Injury and New Technologies (SPRINT) Research Centre, Australian Catholic University, Brisbane, Queensland, Australia; School of Behavioural and Health Sciences, Australian Catholic University, Brisbane, Australia; Sports Performance, Recovery, Injury and New Technologies (SPRINT) Research Centre, Australian Catholic University, Brisbane, Queensland, Australia; School of Behavioural and Health Sciences, Australian Catholic University, Brisbane, Australia; Sports Performance, Recovery, Injury and New Technologies (SPRINT) Research Centre, Australian Catholic University, Brisbane, Queensland, Australia

**Keywords:** adenosine, sleepiness, sleep disruption, sleep behaviors, sleep recommendations

## Abstract

**Study Objectives:**

To investigate the effect of a typical dose of caffeine and a high dose of caffeine consumed in the morning, afternoon, and evening on subsequent sleep.

**Methods:**

Using a placebo-controlled, double-blind, randomized crossover design, 23 males (25.3 ± 5.0 years) with a moderate habitual caffeine intake (<300 mg∙day^−1^) completed seven conditions: placebo, and 100 and 400 mg of caffeine consumed 12, 8, and 4 hours prior to bedtime, with a 48-hour washout. In-home partial polysomnography and sleep diaries were used to assess sleep. Linear mixed models estimated the effect of each condition.

**Results:**

No significant effect on objective or subjective sleep occurred with the 100 mg dose of caffeine compared with the placebo (*p* > .05), but significant effects occurred with the 400 mg dose (*p* < .05). Significant delays in sleep initiation and alterations to sleep architecture were observed when 400 mg was consumed within 12 hours of bedtime (*p* < .05), and significantly greater sleep fragmentation occurred when 400 mg was consumed within 8 hours of bedtime (*p* < .05). Additionally, perceived sleep quality was significantly reduced when 400 mg was consumed 4 hours prior to bedtime (−34.02%, *p* = .006) but not at 8 or 12 hours.

**Conclusions:**

A 100 mg dose of caffeine can be consumed up to 4 hours prior to bedtime, but 400 mg may negatively impact sleep when consumed as one dose within 12 hours of bedtime, with the adverse influence on sleep increasing the closer consumption occurs to bedtime. The discrepancy between objective and subjective sleep quality suggests that individuals may have difficulty accurately perceiving the influence of caffeine on sleep quality.

**Clinical trial registration:**

Australian and New Zealand Clinical Trials Registry, registration number: ACTRN12621001625864, https://www.anzctr.org.au/Trial/Registration/TrialReview.aspx?ACTRN=12621001625864.

## Graphical Abstract

**Graphical Abstract:**
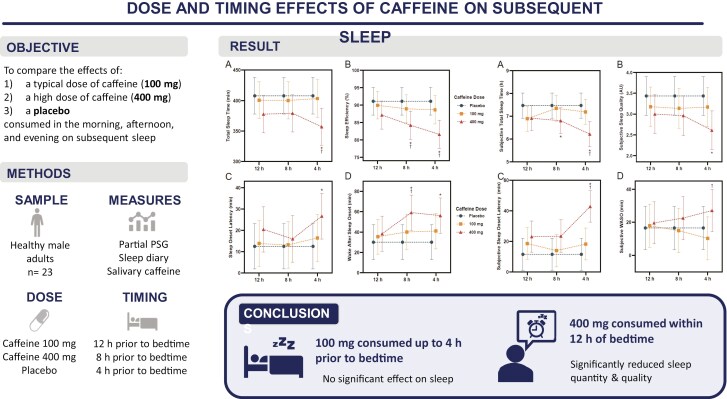

**Statement of Significance**

Current sleep behavior recommendations are insufficient by simply suggesting consumers limit caffeine intake prior to bedtime. This study provides caffeine consumers with cutoff times to minimize negative effects on sleep. Importantly, the findings show that consumers have difficulty perceiving sleep disruption following caffeine consumption and highlight the need for greater consumer education.

Caffeine is a legal psychostimulant consumed by ~80% of the population [1]. The ability of caffeine to elicit positive changes in alertness and cognitive performance contributes to its widespread use [2]. Caffeine acts as an adenosine receptor antagonist to block the action of adenosine, a neuromodulator implicated in the homeostatic regulation of sleep and wake [3]. Sleep pressure is suggested to rise in accordance with the accumulation of extracellular adenosine during wake and reduce with its dissipation during sleep [4]. As such, caffeine can be employed as a strategy to offset high sleep pressure experienced during periods of insufficient sleep [5]. It is estimated that up to 40% of the population fail to obtain sufficient sleep [6–9], highlighting the utility of caffeine as a strategy to minimize the detrimental effects associated with insufficient sleep. However, the consumption of caffeine may interfere with subsequent sleep creating a potential cycle of poor sleep and reliance on caffeine [10].

Evening caffeine consumption typically reduces the quantity and quality of subsequent sleep [11]. Experimental studies show that 100-600 mg of caffeine can significantly increase sleep onset latency (SOL) [12–23] and wake after sleep onset (WASO) [12, 15, 20, 21, 24, 25] with reductions in total sleep time (TST) [12, 13, 15–26] and sleep efficiency (SE) [12, 14–25]. Of these studies outlined, four involved the administration of multiple caffeine doses, demonstrating a greater influence on sleep when larger doses were consumed [12, 17, 23, 27]. Additionally, caffeine doses ranging from 300-600 mg increase the occurrence of non-rapid eye movement (NREM) stages 1 (N1) and 2 (N2) (i.e. light sleep) with reductions in the occurrence of NREM stage 3 (N3) (i.e. deep sleep) [12, 13, 17, 23–26], although this effect is not consistent when doses of 200 mg or less [14, 16–19, 21–24] are consumed. Collectively, the current evidence suggests a dose-dependent impact of caffeine on subsequent sleep, whereby larger doses of caffeine may result in greater sleep disruptions.

The timing of caffeine consumption is another key consideration when assessing the impact of caffeine on subsequent sleep. Sleep disruption is consistently reported when caffeine is consumed within 3 hours of bedtime [12–14, 16–18, 21–25], with inconsistent effects when caffeine is administered in the morning or afternoon [15, 19, 26, 28, 29]. In accordance with the current evidence, sleep behavior recommendations commonly promote the avoidance of caffeine close to bedtime to mitigate sleep disruption [30, 31]. Given evening caffeine consumption represents only a small portion of daily caffeine intake, it is necessary to consider the typical patterns of habitual consumers when evaluating the effect of caffeine on subsequent sleep [32]. Additionally, there is substantial individual variability in the half-life and effect of caffeine, largely due to genetic predispositions, which should be considered when investigating the influence of caffeine dose and timing of intake on sleep [33]. To provide specific guidance regarding the dose of caffeine and the appropriate time of day to discontinue intake, experimental investigations of the combined influence of the *dose* and *timing* of caffeine consumption on subsequent sleep are required. Therefore, the aim of this study is to investigate the effect of varying caffeine dose and timing combinations on the characteristics of subsequent sleep.

## Materials and Methods

### Participants

Based on a repeated-measures, within-subject design, a sample size calculation was performed using G*Power statistical software. Based on a small effect size difference (Cohen’s *f* = 0.2), an alpha level of 0.05, and 80% power, a sample size of 21 participants was required based on the primary outcome variable of TST. Due to the repeated measurements within individuals, a correlation of 0.6 [23] was used based on raw data from a recent meta-analysis [11]. To be included, participants were required to be healthy males aged between 18 and 40 years with a moderate habitual caffeine intake (<300 mg∙day^−1^) assessed using a validated self-report tool [34]. Females were excluded from this study due to the timeframe required to account for differing hormonal profiles, including menstrual cycle phase for naturally cycling females, which would have extended the protocol and potentially introduced confounding variables over time. General health was assessed using the 12-Item General Health Questionnaire (GHQ-12) [35], with participants considered eligible if 80% or more of their responses met the criteria. Sleep quality was assessed using the Pittsburgh Sleep Quality Index (PSQI) [36] with participants considered eligible if they scored ≤5. Participants were excluded if they reported a sleep or medical condition and consumed cigarettes, drugs, or medications known to affect sleep within the 3 months prior to study admission, or if they had undertaken shift work or transmeridian travel within the 3 months prior to study admission [37]. Finally, due to the potential influence of genetic predispositions on caffeine metabolism and sensitivity, genetic testing of single nucleotide polymorphisms (SNPs) in the *cytochrome P450 1A2* (*CYP1A2*) variant rs762551 and *adenosine A2A receptor* (*ADORA2A*) variant rs5751876 was conducted for each participant and is provided in Supplementary Table 1. These data provide additional context which is an important consideration when completing caffeine research. Each participant provided informed, written consent and received financial compensation for their participation in the study.

### Experimental design and protocol

The study was a placebo-controlled, double-blind, randomized crossover design approved by the Australian Catholic University (ACU) Human Research Ethics Committee (2021-233HC) and registered with the Australian and New Zealand Clinical Trials Registry (ACTRN12621001625864). Participants completed seven conditions separated by a 48-hour washout period to account for the half-life of caffeine (typically 3–6 hours [38]) and allow potential sleep disruptions to dissipate between conditions. The caffeine conditions included two different doses of caffeine (100 or 400 mg) administered once across three different time points (12, 8, or 4 hours prior to bedtime). The 100 mg dose represents a typical dose of caffeine (approximately one cup of coffee [39]), while the 400 mg dose represents a high dose of caffeine (upper recommended daily limit [40]). The control condition involved the placebo being administered at each of the three timepoints. Conditions were administered using identical appearing and weight-matched gelatin capsules containing caffeine anhydrous (Merck, Germany) or glucose powder (iNova Pharmaceuticals, Australia). The order of administration was organized using a Latin Square design constructed using a random allocation schedule (i.e. random sequence of A to G) generated through a random number sequence (i.e. 1 = A through to 7 = G) (RANDOM.ORG, Ireland). Each participant was randomized into a condition sequence upon enrollment in the study using block randomization, where each block consisted of seven potential sequences. The generation and allocation of the sequence of interventions was performed by an independent research team member who did not interact with participants or assist in data analysis. The protocol included a 7-day baseline monitoring period followed by a 21-day intervention period, with no change to the protocol or outcomes of interest after trial commencement. All testings were performed in the participants’ home environments.

#### Baseline monitoring

Participants wore an activity monitor (Spectrum Plus; Phillips Respironics, United States) and completed a daily electronic sleep diary via the Research Electronic Data Capture (REDCap) tool hosted at ACU for 7 consecutive days [41]. During this baseline period, participants were instructed to aim for consistency around their typical bed and wake time (±30 minutes). The data from this baseline monitoring were averaged to calculate each participant’s habitual bed and wake times, which were then used to individualize the intervention protocol. Participant compliance with the individualized intervention schedule is provided in Table 1. Additionally, participants completed a validated caffeine consumption questionnaire [34] to quantify daily habitual caffeine intake. To control for and standardize caffeine intake [42, 43], participants consumed a capsule containing their habitual caffeine intake within 1 hour of waking on each day of the intervention and refrained from consuming additional sources of caffeine for the duration of the intervention, with compliance monitored through a diet diary (MyNetDiary, United States).

**Table 1.** Participant compliance with scheduled bedtime and wake time on condition days

Proximity to individualized schedule	Bedtime	Wake time
≤30 minutes	82.8%	78.3%
>30 to ≤45 minutes	7.6%	7.0%
>45 to ≤60 minutes	6.4%	4.5%
>60 to ≤75 minutes	2.6%	6.4%
>75 to ≤90 minutes	0%	2.5%
>90 to ≤105 minutes	0%	1.3%
>105 to ≤120 minutes	0.6%	0%

#### Intervention period

**Figure 1.**
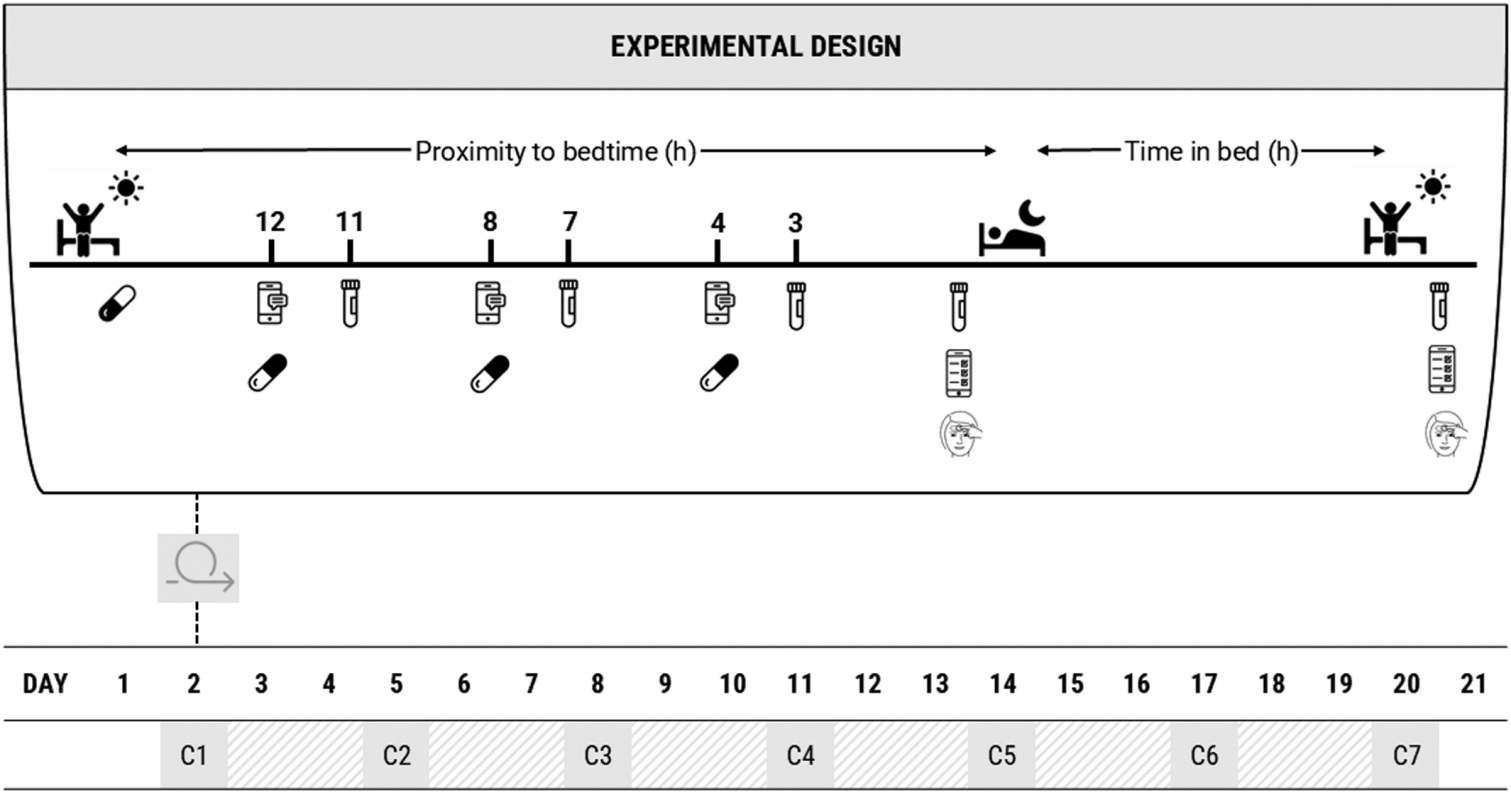
Experimental phase of the study protocol. Participants completed seven conditions (C1–C7) in a randomized manner with a 48-hour washout period between conditions. On each condition day, participants consumed their habitual daily caffeine consumption within 1 hour of waking in capsule form. On condition days, three capsules (placebo or caffeine) were administered at 12, 8, and 4 hours prior to bedtime in line with the assigned condition. Saliva samples were taken 1-hour post-consumption at 11, 7, and 3 hours prior to bedtime, with additional saliva samples taken 5 minutes prior to bedtime and 5 minutes post-waking. Immediately prior to bedtime, participants applied the Somfit device and completed the Karolinska Sleepiness Scale. Upon waking, participants removed the Somfit device, completed the sleep diary, and recorded their perception of condition.

Figure 1 displays a schematic outlining the intervention phase. Participants were required to maintain a consistent bed and wake time (±30 minutes) and refrain from napping throughout the intervention. As per the baseline period, participants wore an activity monitor and completed a sleep diary and diet diary daily to monitor compliance. On each condition day, three capsules were administered at 12, 8, and 4 hours prior to bedtime. Habitual bedtimes derived from the baseline monitoring were used to calculate individualized time points of consumption for each participant. For the caffeine conditions, one capsule contained caffeine (100 or 400 mg) with the remaining two capsules containing the placebo. For the control condition, all capsules contained the placebo. Each participant was provided a capsule case with the order, time, and day of each capsule’s consumption clearly identified. Additionally, on each condition day, participants received a phone text message to remind them to consume the specified capsule with compliance being confirmed with a return message.

### Measures

#### Salivary caffeine concentration

Saliva samples were collected 60 minutes after each capsule (11, 7, and 3 hours prior to bedtime), 5 minutes prior to bedtime, and 5 minutes post-waking using Salivette devices (Sarstedt, Germany). To ensure sample integrity, participants were provided with a portable freezer (Dometic CF11; Dometic Group, Sweden) to store the collected samples at −18°C until the conclusion of the protocol. Samples were tested in duplicate for caffeine levels using enzyme-linked immunosorbent assay (ELISA) analysis performed according to the manufacturer’s protocol (ab285229; Abcam, Australia).

#### Objective sleep

Sleep was assessed using the Somfit (Compumedics, Australia), a validated partial polysomnography device [44]. Participants attached the device to their forehead prior to bedtime using a disposable electrode patch and controlled the overnight recording via the Somfit smartphone application. The recorded data were subsequently uploaded to the Compumedics server and analyzed using a proprietary algorithm (Compumedics, Australia). The following dependent variables were calculated for each sleep record: TST; SE (TST/time in bed × 100); SOL (time from lights out to first sleep epoch); latency to persistent sleep (time from lights out to first 10 minutes of consecutive sleep epochs); rapid eye movement (REM) SOL (time from lights out to first epoch of REM sleep); WASO (time spent awake following sleep onset); number of awakenings (transition from a sleep epoch to a wake epoch) per hour; absolute sleep architecture (duration of time spent in each stage of N1 and N2 sleep [light sleep], N3 sleep, and REM sleep); and relative sleep architecture (proportion of time spent in each stage of N1 and N2 sleep [light sleep], N3 sleep, and REM sleep relative to TST).

#### Subjective sleep

Subjective sleep was assessed using an electronic sleep diary. Participants completed the sleep diary within 30 minutes of waking to provide dependent variables of subjective TST (hours) asked as “how long did you sleep in total last night?,” SOL (minutes) asked as “how long did it take you to fall asleep last night,” WASO (minutes) asked as “after falling asleep last night, how long did you spend awake (rather than sleeping) before getting up in the morning,” sleep quality (1–5 Likert scale anchored by “1” as equivalent to “very poor” and “5” equivalent to “very good”), and presleep alertness (1–5 Likert scale anchored by “1” as equivalent to “none” and “5” equivalent to “very high”). Participants indicated their acute level of sleepiness immediately prior to bedtime using the Karolinska Sleepiness Scale (1–9 Likert scale anchored by “1” as equivalent to “extremely alert” and “9” equivalent to “very sleepy, great effort to keep alert, fighting sleep”).

#### Perception of condition

On the morning following a condition, participants completed an electronic questionnaire within 30 minutes of waking to indicate whether they believe that they had consumed caffeine, and if so, at what dose they believe this occurred.

#### Genotyping of SNPs

Each participant provided a saliva sample with genomic deoxyribonucleic acid (DNA) isolated using an extraction kit (QIAsymphony DSP DNA Midi Kit; Qiagen, Germany) following the manufacturer’s recommendations. Genotypes for SNPs rs762551 in *CYP1A2* and rs5751876 in *ADORA2A* were assessed in all participants and selected in accordance with reported genetic associations to individual variability in caffeine half-life and effect [33, 45–49]. SNP genotyping was performed using the Multiplex MassARRAY genotyping assay (Sequenom, Inc., United States), with the analysis performed by matrix-assisted laser desorption/ionization time-of-flight mass spectrometry.

### Statistical analysis

All statistical analyses were performed in RStudio (Posit, United States) using the R programming language (Version 4.2.2 *Innocent and Trusting*, R Foundation for Statistical Computing, Austria) and associated packages. To determine the impact of caffeine on sleep outcomes and salivary caffeine concentrations, linear mixed models were built using the *lmerTest* package and *lmer* function. All sleep models were fit with restricted estimated maximum likelihood, participant ID was included as a random effect and the caffeine condition was included as a fixed effect. The salivary caffeine concentrations were assessed with a time × condition interaction included as fixed effects. Whilst mixed models are robust to non-normally distributed residuals, the residuals from each model were plotted and inspected to ensure that they were somewhat normally distributed [50]. Estimated marginal means post-hoc analyses were performed to determine differences between conditions using the *emmeans* and *contrasts* functions from the *emmeans* package. Five sets of 5 contrasts (15 contrasts per analysis) were created to compare each dose within each timepoint (three sets of 3 contrasts) and each timepoint within each dose (three sets of 2 contrasts). A Bonferroni familywise adjustment was performed to account for the multiple comparisons within each set of contrasts. The magnitude of differences was interpreted by estimating appropriately weighted Cohen’s *d* effect sizes (*d*) and 95% confidence intervals (CIs) from the *t*-statistic from the mixed models using the *t_to_d* function from the *effectsize* package. Effect sizes were interpreted as *trivial* <0.20; *small*, 0.20–0.49; *medium*, 0.50–0.79; and *large* ≥0.8. Adjusted statistical significance was set to *p* < .050 and all data are reported as means ± standard deviation (*SD*).

## Results

### Participants

A sample of 30 eligible participants volunteered to participate in the study between June 2022 and March 2023. Following randomization, three participants withdrew prior to starting the intervention due to the required time commitment, and four participants were excluded from the analysis due to noncompliance with the protocol (Supplementary Figure 1). The trial ended in June 2023 upon satisfying the required sample size, with a final sample of 23 participants included in the analysis. The baseline demographics of the study sample are reported in Table 2. There were no adverse events (i.e. no unintended harms) reported throughout the study period.

**Table 2.** Baseline demographics of study sample

	Sequence Allocation							
	DCAGEBF (*n* = 3)	CAGEBFD (*n* = 4)	AGEBFDC (*n* = 3)	GEBFDCA (*n* = 2)	EBFDCAG (*n* = 3)	BFDCAGE (*n* = 4)	FDCAGEB (*n* = 4)	Total (*n* = 23)
Age (years)	25.0 ± 5.6	26.8 ± 2.1	20.3 ± 1.5	24.5 ± 0.7	30.3 ± 9.5	26.3 ± 4.9	23.3 ± 3.2	25.3 ± 5.0
Mass (kg)	89.9 ± 18.1	81.3 ± 14.4	83.7 ± 23.7	88.8 ± 15.3	89.0 ± 13.5	84.8 ± 29.9	84.1 ± 8.3	85.5 ± 16.8
Height (cm)	183.7 ± 11.8	182.5 ± 12.2	183.3 ± 1.5	176.5 ± 4.9	177.0 ± 6.6	181.8 ± 10.0	179.0 ± 11.2	180.1 ± 8.5
Habitual caffeine (mg∙day^−1^)	191.6 ± 103.3	72.7 ± 40.5	80.0 ± 8.6	120.4 ± 14.9	122.1 ± 154.9	129.8 ± 110.0	87.1 ± 19.1	112.2 ± 81.0
PSQI (score)	3.0 ± 2.0	3.3 ± 1.7	4.0 ± 1.0	3.5 ± 2.1	3.0 ± 1.0	3.8 ± 0.5	2.8 ± 0.5	3.3 ± 1.2
GHQ-12 (%)	97.2 ± 4.8	89.6 ± 8.0	97.2 ± 4.8	91.7 ± 11.8	94.4 ± 4.8	89.6 ± 8.0	100.0 ± 0.0	94.2 ± 6.9

### Salivary caffeine concentration

**Figure 2.**
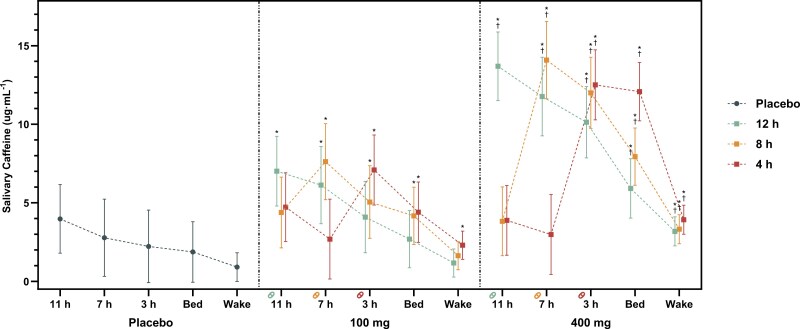
Salivary caffeine concentration (µg∙mL^−1^) measured 11 (60 minutes after capsule 1), 7 (60 minutes after capsule 2), and 3 hours (60 minutes after capsule 3) prior to bedtime, 5 minutes prior to bedtime, and 5 minutes post-waking the following morning for each condition. *Indicates a significant difference to the placebo at the same timepoint; ^†^Indicates a significant difference compared with the 100 mg dose of caffeine at the same timepoint. Error bars represent adjusted 95% CIs.

Figure 2 presents salivary caffeine concentrations at each sampling time with comparisons detailed in Supplementary Table 2. For measures taken 60 minutes post-consumption, salivary caffeine concentrations were significantly higher than the placebo for each condition (*p* < .050). For each timepoint of consumption, the 400 mg dose of caffeine resulted in significantly higher salivary caffeine concentrations compared with the 100 mg dose of caffeine (*p* < .050).

### Objective sleep

**Figure 3.**
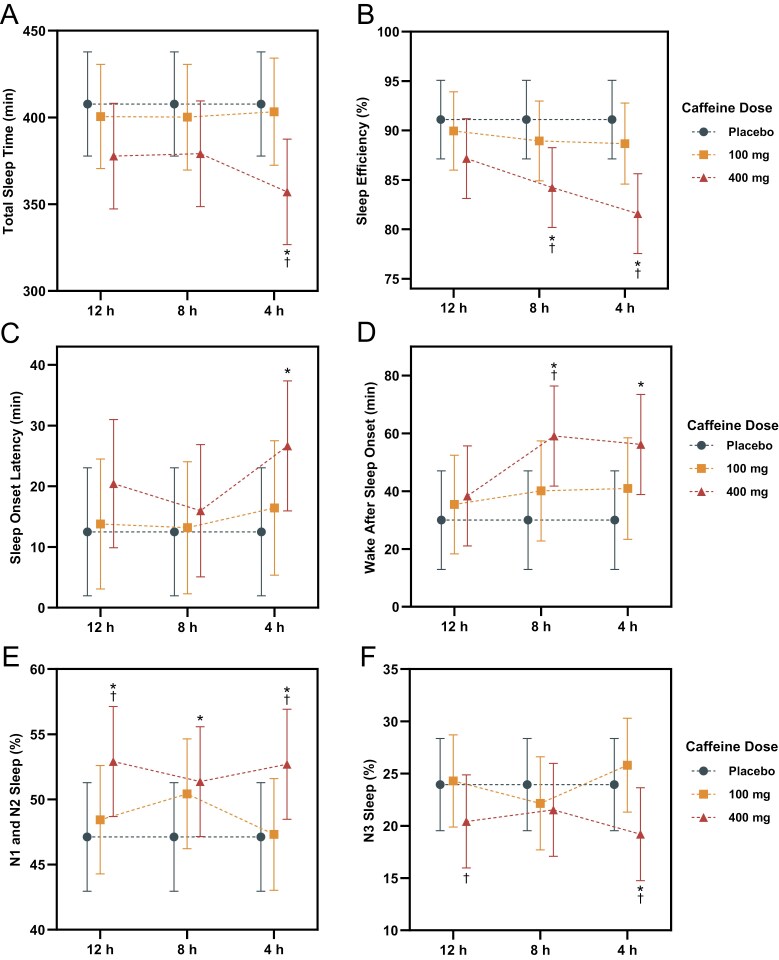
Displays the change in the objective sleep outcomes of (A) TST (minutes); (B) SE (%); (C) SOL (minutes); (D) WASO (minutes); (E) proportion of N1 and N2 sleep (%); and (F) proportion of N3 sleep (%) for placebo, 100 mg of caffeine, and 400 mg of caffeine consumed 12, 8, and 4 hours prior to bedtime. *Indicates a significant difference to the placebo within the same timepoint.; ^†^Indicates a significant difference compared with the 100 mg dose of caffeine within the same timepoint. Error bars represent adjusted 95% CIs.

Figure 3 displays objective sleep outcomes with descriptive statistics detailed in Supplementary Table 3 and comparisons in Supplementary Table 4. Table 1 outlines the compliance rates of participants with their scheduled bed and wake times. No significant difference was observed in bedtime, waketime, or time in bed between conditions. The 400 mg dose of caffeine significantly affected objective sleep compared with the placebo. TST was reduced by an estimated 50.6 minutes (*p* < .001; *d* = −0.36 [−0.59 to −0.14]) when 400 mg was consumed 4 hours prior to bedtime, with a small, nonsignificant effect when consumed at 8 (estimate: −28.7 minutes; *p* = .076; *d* = −0.21 [−0.43 to 0.02]) and 12 hours (estimate: −30.0 minutes; *p* = .060; *d* = −0.22 [−0.44 to 0.01]). SE was reduced by an estimated 9.5% (*p* < .001; *d* = −0.48 [−0.71 to −0.25]) when 400 mg was consumed 4 hours prior to bedtime and by 6.9% (*p* = .001; *d* = −0.35 [−0.58 to −0.12]) when consumed 8 hours prior to bedtime. SOL increased by an estimated 14.2 minutes (*p* = .013; *d* = 0.26 [0.04–0.48]) when 400 mg was consumed 4 hours prior to bedtime. For SOL to persistent sleep, there was an estimated increase of 25.4 minutes (*p* < .001; *d* = 0.41 [0.18–0.63]) and 15.3 minutes (*p* = .020; *d* = 0.25 [0.03–0.47]) when 400 mg was consumed 4 and 12 hours prior to bedtime, respectively. WASO was increased by an estimated 26.2 minutes (*p* = .002; *d* = 0.32 [0.09–0.54]) when 400 mg was consumed 4 hours prior to bedtime and by 29.1 minutes (*p* = .001; *d* = 0.36 [0.13–0.58]) when consumed 8 hours prior to bedtime. In addition, there was an estimated increase of 1.4 awakenings per hour (*p* = .001; *d* = 0.33 [0.10–0.55]) when 400 mg was consumed 4 hours prior to bedtime. The proportion of N1 and N2 sleep increased by an estimated 5.6% (*p* = .007; *d* = 0.28 [0.06–0.51]) when 400 mg was consumed 4 hours prior to bedtime and by 5.8% (*p* = .005; *d* = 0.30 [0.07–0.52]) when consumed 12 hours prior to bedtime, with a nonsignificant, small effect (+4.2%; *p* = .056; *d* = 0.22 [0.00–0.44]) at 8 hours. With the 400 mg dose of caffeine, the duration of N3 sleep was reduced by an estimated 29.7 minutes (*p* < .001; *d* = −0.50 [−0.73 to −0.27]) when consumed 4 hours prior to bedtime, by 15.3 minutes (*p* = .016; *d* = −0.26 [−0.48 to −0.04]) when consumed 8 hours prior to bedtime, and by 20.6 minutes (*p* = .001; *d* = -0.35 [−0.57 to −0.12]) when consumed 12 hours prior to bedtime. Additionally, the proportion of N3 sleep was reduced by an estimated 4.8% (*p* = .005; *d* = −0.30 [−0.52 to −0.07]) when 400 mg was consumed 4 hours prior to bedtime. No significant effects were observed on objective sleep outcomes with consumption of the 100 mg dose of caffeine (*p* > .050).

#### Dose contrasts

The 400 mg dose of caffeine had a greater impact on sleep than the 100 mg dose. TST was reduced by an estimated 46.2 minutes (*p* = .002; *d* = −0.32 [−0.55 to −0.10]) when 400 mg was consumed 4 hours prior to bedtime compared with 100 mg. SE was reduced by an estimated 7.1% (*p* = .001; *d* = −0.35 [−0.57 to −0.12]) when 400 mg was consumed 4 hours prior to bedtime and by an estimated 4.7% (*p* = .034; *d* = −0.23 [−0.46 to −0.01]) when consumed 8 hours prior to bedtime compared with 100 mg at each respective timepoint. When consuming 400 mg as opposed to 100 mg, latency to persistent sleep was increased by an estimated 17.5 minutes (*p* = .010; *d* = 0.27 [0.05–0.49]) when consumed at 4 hours and WASO was increased by an estimated 19.0 minutes (*p* = .043; *d* = 0.23 [0.01–0.45]) when consumed at 8 hours. With the 400 mg dose of caffeine compared with the 100 mg dose of caffeine, the proportion of N1 & N2 sleep increased by an estimated 5.4% (*p* = .012; *d* = 0.27 [0.04–0.49]) and 4.5% (*p* = .041; *d* = 0.23 [0.01–0.45]) with consumption at 4 and 12 hours prior to bedtime, respectively. Furthermore, the duration and proportion of N3 sleep was reduced by an estimated 33.8 minutes (*p* < .001; *d* = −0.56 [−0.79 to −0.32]) and 6.6% (*p* < .001; *d* = −0.40 [−0.63 to −0.17]) when consumed 4 hours prior to bedtime and by 20.0 minutes (*p* = .001; *d* = −0.34 [−0.56 to −0.11]) and 3.9% (*p* = .028; *d* = −0.24 [−0.46 to −0.02]) when consumed 12 hours prior to bedtime.

#### Timing contrasts

The timing of the 400 mg dose of caffeine influenced the effect on subsequent sleep. SE was reduced by an estimated 5.6% (*p* = .008; *d* = −0.28 [−0.51 to −0.06]) when 400 mg was consumed 4 hours prior to bedtime compared with 12 hours. WASO was increased by an estimated 20.8 minutes (*p* = .023; *d* = 0.25 [0.03–0.47]) when 400 mg was consumed 8 hours prior to bedtime compared with 12 hours. Additionally, an estimated increase of 1.0 awakening per hour (*p* = .035; *d* = 0.24 [0.01–0.46]) was observed when 400 mg was consumed 4 hours prior to bedtime compared with 12 hours. No significant effects were observed for the consumption of the 100 mg dose of caffeine across each timepoint (*p* > .050).

### Subjective sleep

**Figure 4.**
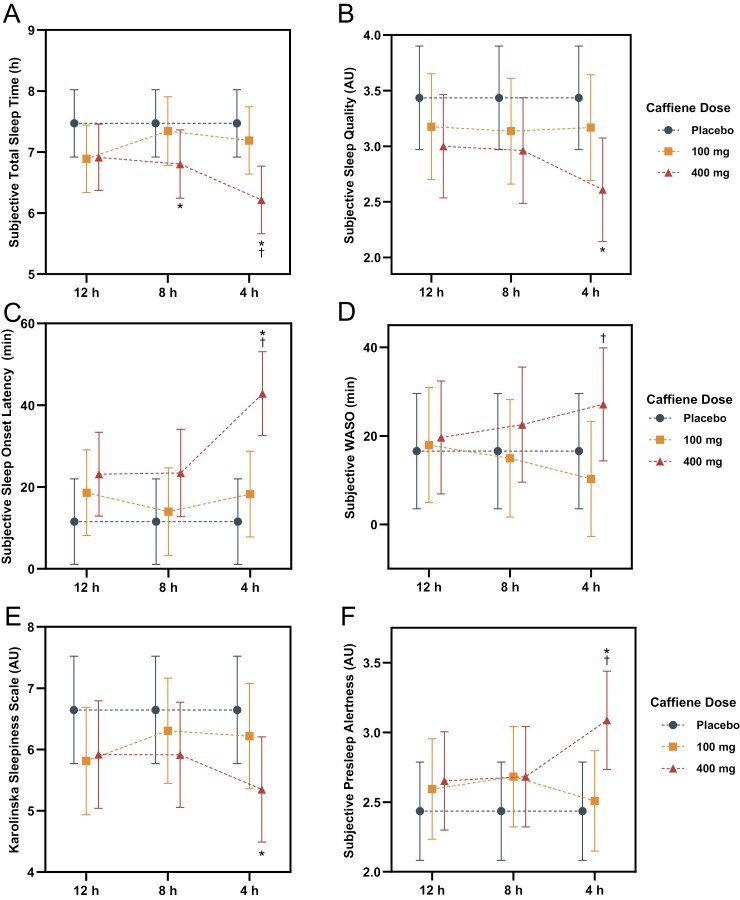
Displays the change in the subjective sleep outcomes of: (A) TST (hours); (B) sleep quality (units); (C) SOL (minutes); (D) WASO (minutes); (E) Karolinska Sleepiness Scale (units); and (F) presleep alertness (units) for placebo, 100 mg of caffeine, and 400 mg of caffeine consumed 12, 8, and 4 hours prior to bedtime. *Indicates a significant difference to the placebo within the same timepoint; ^†^Indicates a significant difference compared with the 100 mg dose of caffeine within the same timepoint. Error bars represent adjusted 95% CIs.

Figure 4 displays subjective sleep outcomes with descriptive statistics detailed in Supplementary Table 3 and comparisons in Supplementary Table 5. The 400 mg dose of caffeine reduced perceived TST by an estimated 1.3 hours (*p* < .001; *d* = −0.44 [−0.66 to −0.22]) when consumed 4 hours prior to bedtime and by 0.7 hours (*p* = .034; *d* = −0.23 [−0.45 to −0.01]) when consumed 8 hours prior to bedtime, with a nonsignificant, small effect at 12 hours (estimate: 0.6 hours, *p* = .087; *d* = −0.20 [−0.42 to 0.02]). Additionally, when consumed 4 hours prior to bedtime, 400 mg increased presleep alertness by an estimated 0.7 units (*p* = .003; *d* = 0.30 [0.08–0.51]) and perceived SOL by 31.3 minutes (*p* < .001; *d* = 0.50 [0.27–0.72]) while reducing sleepiness by 1.3 units (*p* = .019; *d* = −0.24 [−0.46 to −0.03]) and perceived sleep quality by 0.8 units (*p* = .006; *d* = −0.28 [−0.50 to −0.06]). The 100 mg dose of caffeine had no significant effect on subjective sleep outcomes (*p* > .050).

#### Dose contrasts

Dose contrasts revealed that the 400 mg dose of caffeine compared with the 100 mg dose had a greater impact on subjective sleep when consumed 4 hours prior to bedtime, reducing perceived TST by an estimated 1.0 hours (*p* = .001; *d* = −0.34 [−0.56 to −0.12]), increasing perceived SOL by an estimated 24.6 minutes (*p* < .001; *d* = 0.39 [0.17–0.61]), and increasing perceived WASO by an estimated 16.9 minutes (*p* = .037; *d* = 0.23 [0.01–0.44]). No significant dose effects were observed with consumption eight or 12 hours prior to bedtime (*p* > .050).

#### Timing contrasts

Timing contrasts revealed that the impact of the 400 mg dose of caffeine was influenced by the timing of consumption. When 400 mg was consumed 4 hours prior to bedtime, perceived SOL increased by an estimated 19.4 minutes (*p* = .003; *d* = 0.30 [0.09–0.52]) and 19.7 minutes (*p* = .002; *d* = 0.32 [0.10–0.54]) compared with 8 and 12 hours, respectively. Additionally, TST was reduced by an estimated 0.7 hours (*p* = .020; *d* = −0.25 [−0.47 to −0.03]) compared with consumption at 12 hours. No timing effect was observed for the 100 mg dose of caffeine (*p* > .050).

### Perception of condition

When asked to state which dose of caffeine and the timing of that dose they received, 44% of participants identified the correct dose, 36% identified the correct timing of consumption, and 22% concurrently identified the correct dose and timing across conditions.

### Genotyping for rs762551 and rs5751876

The genotypic distribution for the tested SNPs was assessed for deviation from the Hardy–Weinberg Equilibrium. No significant deviations (*p* > .050) were observed and the measured genotype frequencies in the sample were consistent with the reported frequencies in the genetic database for the Caucasian cohort (Supplementary Table 1). There was no significant main effect of genotype on objective or subjective sleep outcomes for the SNPs of interest (*p* > .050).

## Discussion

**Figure 5.**
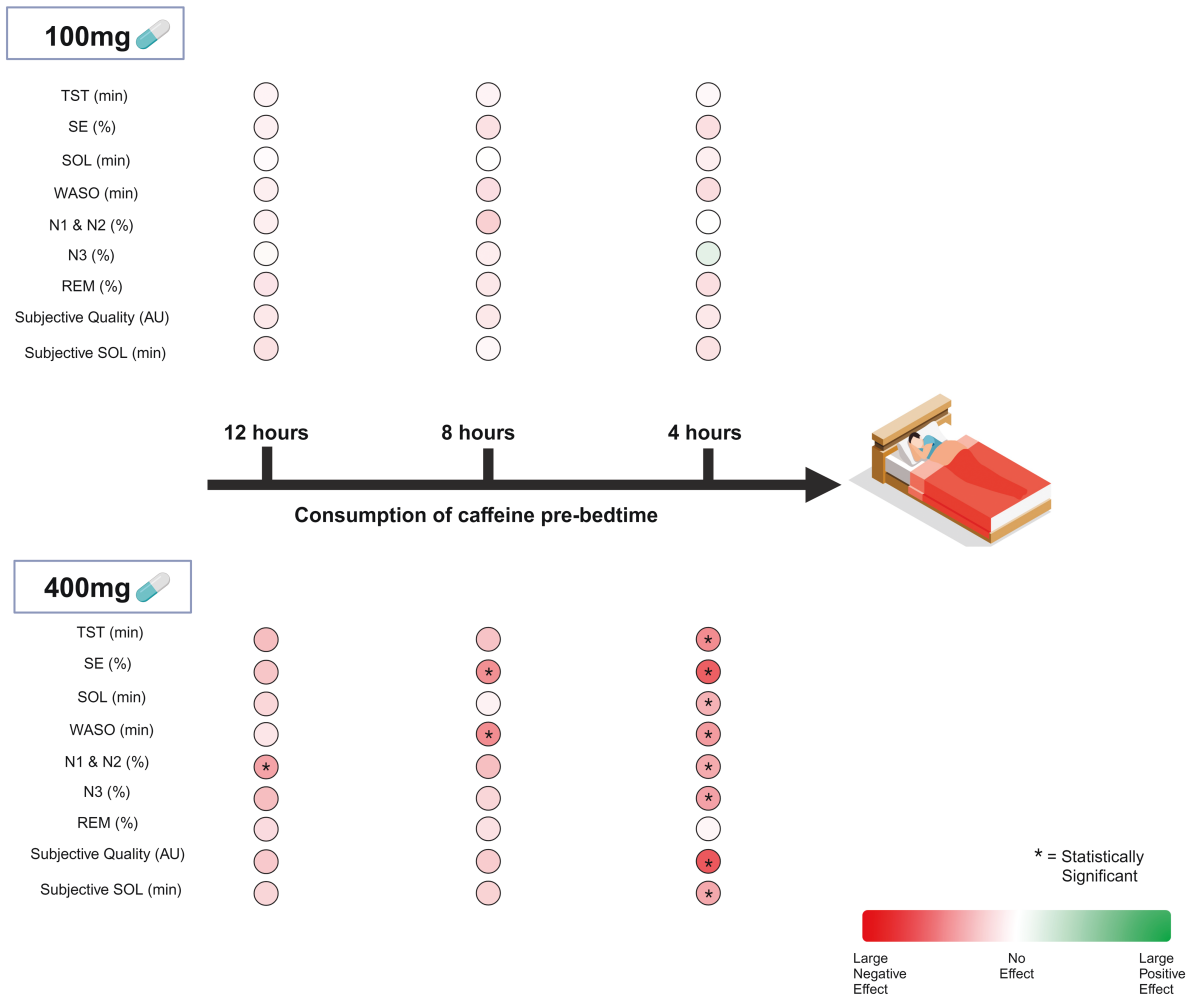
A summary of the effects of 100 mg of caffeine and 400 mg of caffeine on TST, SE, SOL, WASO, NREM stages 1 (N1) and 2 (N2) sleep, NREM stage three (N3) sleep, REM sleep, subjective sleep quality, and subjective SOL when consumed 12, 8, and 4 hours prior to bedtime compared with the placebo. The effect size scale ranges from −0.8 to 0.8 (large effect), with a negative effect indicating a negative influence on the sleep outcome and a positive effect indicating a positive influence on the sleep outcome. *Indicates a statistically significant effect compared with the placebo.

The aim of this study was to investigate the influence of the dose of caffeine and timing of intake on subsequent sleep. Participants consumed 100 and 400 mg doses of caffeine 12, 8, and 4 hours prior to bedtime, with the conditions designed to replicate a typical dose of caffeine and a high dose of caffeine consumed in the morning, afternoon, and evening. A 100 mg dose of caffeine can be consumed up to 4 hours prior to bedtime without a significant effect on subsequent sleep. However, a 400 mg dose of caffeine will significantly delay sleep initiation and alter sleep architecture when consumed within 12 hours of bedtime, with significantly greater sleep fragmentation when consumed within 8 hours of bedtime. Importantly, 400 mg of caffeine negatively impacted perceptions of sleep quantity and quality when consumed 4 hours prior to bedtime, but not when consumed 8 or more hours away from bedtime. A high dose of caffeine (400 mg) consumed as one dose in the morning is detrimental to subsequent night-time sleep, with greater disruptions the closer consumption occurs to bedtime. A typical dose of caffeine (100 mg) can be consumed up to 4 hours prior to bedtime without significant effect on subsequent sleep. To mitigate caffeine-induced sleep disruptions, it is recommended to refrain from consuming 400 mg of caffeine within 12 hours of bedtime (Figure 5).

Initiation and maintenance of sleep were disrupted with consumption of 400 mg of caffeine but not 100 mg. Similar impacts on sleep have been observed when high doses of caffeine (300–600 mg) are consumed within 3 hours of bedtime [12, 13, 15, 17, 25]. As homeostatic sleep pressure increases with the accumulation of cerebral extracellular adenosine, the action of caffeine as an adenosine antagonist can increase alertness and promote wakefulness [51]. Importantly, the changes observed in the present study with 400 mg of caffeine exceed the thresholds for clinically meaningful change in SOL (>10 minutes) when consumed within 12 hours of bedtime and in WASO (>20 minutes) and *SE* (>5%) when consumed within 8 hours of bedtime [52]. On average, consumption of 400 mg within 12 hours of bedtime had a clinically meaningful, small effect on TST (>20-minute threshold), although this effect was only significant when consumed 4 hours prior to bedtime. Consequently, if consumers want to mitigate the negative effect of caffeine on subsequent sleep, 100 mg of caffeine can be consumed up to 4 hours prior to bedtime, but 400 mg of caffeine should not be consumed within 12 hours of bedtime.

Significant alterations in sleep architecture occurred with the 400 mg dose of caffeine but not the 100 mg dose of caffeine. In prior control trials, similar changes in sleep architecture have been reported when 300–600 mg of caffeine has been administered within 6 hours of bedtime [12, 13, 15, 17, 23–25], with inconsistent findings when lower doses (100–150 mg) of caffeine are consumed [18, 22, 24]. N3 sleep is linked to homeostatic sleep regulation, with a tendency for greater N3 sleep when sleep pressure is high [53, 54]. Given that N3 sleep plays a crucial role in physiological restoration, it is largely conserved even when sleep is restricted to 4–7 hours per night [55]. Therefore, the findings of the present study highlight the potent ability of caffeine to reduce N3 sleep. Considering the sleep architecture of a healthy young adult (18–25 years) is comprised of upward of ~50% N1 and N2 sleep and 16%–20% N3 sleep [56], and the tendency for N3 sleep to decline with age [57], the consumption of 400 mg of caffeine within 4 hours of bedtime will markedly reduce the proportion of N3 sleep in healthy males aged 18–40 years. A 100 mg dose of caffeine can be consumed up to 4 hours prior to bedtime, but 400 mg of caffeine should be avoided within 12 hours of bedtime to prevent negative effects on sleep architecture.

Caffeine negatively affected perceptions of sleep in a dose-dependent manner. Significant reductions in perceived TST were reported with consumption of the 400 mg dose of caffeine 4 and 8 hours prior to bedtime. Additionally, SOL increased, and sleep quality decreased when 400 mg was consumed 4 hours prior to bedtime. Reductions in perceived sleep quality were not observed with consumption 8 or 12 hours prior to bedtime. The inability to perceive changes in sleep quality as consumption occurs further away from bedtime is a finding supported by previous work [15]. Interestingly, in the present study, no significant effect on perceived WASO was observed, even though significant increases were detected objectively with consumption 4 and 8 hours prior to bedtime. The discrepancy between objective and subjective measures is consistent with previous investigations, where perceived changes were lacking despite a significant increase in objective WASO [12, 15]. This finding underscores the challenge consumers face in detecting caffeine-induced sleep fragmentation. Furthermore, the findings suggest that consumers can detect negative impacts on sleep when a high dose (400 mg) of caffeine is consumed in close proximity to bedtime. However, the ability to recognize sleep disruption diminishes as consumption occurs further away from bedtime.

When undertaking research that investigates the effects of caffeine, it is important to consider individual variation in metabolism and sensitivity. As identified by Weibel et al. (2021), there is a need to provide greater information regarding participant genotype when evaluating the effect of caffeine on subsequent sleep [28]. The consideration of genetic influence may provide greater context to study outcomes. Functional polymorphisms in the *CYP1A2* variant rs762551 can influence the rate of caffeine metabolism [46, 47, 58, 59], while those in the *ADORA2A* variant rs5751876 can influence caffeine sensitivity [46, 60–62]. Individuals who are sensitive (due to *ADORA2A* genetic variation) or have slow caffeine metabolism (due to *CYP1A2* genetic variation) may experience greater sleep disruption [33, 63]. Consequently, these genotypes were assessed for all participants within this study. However, no statistically significant influence of genotype was identified, although the study was not powered to detect significant effects of genetic susceptibility [64]. Instead, the genotype of participants was included to provide context to the findings in line with best practice recommendations. When visualized as a density plot (Supplementary Figure 2) following the consumption of caffeine, it appeared participants classified as slow metabolizers (i.e. AC/CC genotypes) or sensitive to caffeine (i.e. CT/CC genotype) experienced less TST compared to fast metabolizers (i.e. AA genotype) or those insensitive to caffeine (i.e. TT genotype), respectively. The findings of the present study highlight the need for further research to explore the mediating role of genotype on sleep.

The findings of the present study suggest that moderate habitual caffeine consumers remain susceptible to sleep disruption when consuming a high dose (400 mg) of caffeine. Within the current literature, it is unclear whether habitual consumers develop a tolerance to the sleep-disrupting effects of caffeine [28, 29, 53]. While rodent studies suggest potential adaptations with chronic caffeine exposure, such as increased cerebral adenosine concentrations [65] and upregulated expression of brain adenosine receptors [66–69], the present study indicates that habitual caffeine consumers do not exhibit tolerance to sleep disruption when additional caffeine is consumed at a high dose (400 mg). In a prior study, 450 mg of caffeine was administered to 20 healthy young males for 9 days prior to laboratory testing [28]. Their protocol included 3 doses of 150 mg of caffeine administered 45, 225, and 475 minutes after waking, with the final dose consumed ~8 hours prior to bedtime. There was no significant effect on subsequent sleep measured on the laboratory testing night. Importantly, during the 9 days of in-home caffeine administration preceding the night of laboratory testing, an average delay in sleep onset of 25 minutes was reported when caffeine (450 mg per day) was consumed compared with the placebo, suggesting that participants may have experienced caffeine-induced sleep disruption prior to laboratory testing. Overall, the existence of tolerance to caffeine-induced sleep disturbance remains unclear in habitual consumers.

## Limitations

The present study offers valuable insights into the impact of both the dose of caffeine and the timing of intake on subsequent sleep in a free-living environment. However, several limitations should be considered when interpreting the findings. First, while conducting an in-home study increases ecological validity, there are potential drawbacks including uncontrolled environmental factors (e.g. partners, children, pets, noise, and lighting) that would normally be controlled in a laboratory environment [70]. In addition, dietary factors (e.g. macronutrient composition and timing) were not standardized [71]. Given the diet diary was employed to monitor caffeine intake rather than timing and composition of food intake, dietary intake could not be accounted for in the statistical analysis. Although the randomization protocol aimed to minimize the impact of these factors, there is potential for a confounding influence on subsequent sleep. Furthermore, sleep recorded on a condition day may have been impacted by variation in the duration of prior waking and/or the quantity and quality of sleep the prior night, which was not accounted for in the statistical analysis. Second, the dose of caffeine was administered at a single timepoint, and thus, it remains unclear whether the findings would be influenced by the consumption of the same dose of caffeine across multiple timepoints in the day. Third, the study employed a healthy young adult male population which may limit the generalizability of the findings to alternative populations. For example, older adults (>40 years) have been shown to be susceptible to greater caffeine-induced sleep disruption [16], while a female population may be influenced by differing hormonal profiles, especially with the use of oral contraception which is known to increase the half-life of caffeine [33]. Lastly, although the Somfit is valid for detecting meaningful changes in sleep architecture over time [44], caution should be applied when considering alterations in sleep architecture measured from a single night of assessment for each condition.

## Practical Recommendations

Current sleep behavior recommendations lack specificity regarding the appropriate time to cease caffeine intake prior to bedtime [63]. A previous quantitative synthesis of the literature recommends avoiding a standard cup of coffee (107 mg per 250 mL [39]) within 8.8 hours of bedtime to prevent significant reductions in TST [11]. However, as this cutoff time was established through statistical modelling, the absolute reduction in TST could not be determined to evaluate the clinical significance. The present study extends this research by identifying timepoints for the appropriate consumption of a typical dose of caffeine (100 mg) and a high (400 mg) dose of caffeine. From the findings, it is recommended that a 100 mg dose of caffeine can be consumed up to 4 hours prior to bedtime without a negative impact on subsequent sleep. Conversely, subsequent sleep is negatively impacted when 400 mg of caffeine is consumed 12, 8, and 4 hours prior to bedtime, with greater effects the closer consumption occurs to bedtime. Accordingly, it is recommended to avoid consumption of 400 mg of caffeine as 1 dose within 12 hours of bedtime. When considering common caffeine products, 400 mg equates to approximately four standard coffees (107 mg per 250 mL [39]), three energy drinks (160 mg per 500 mL [72]), or two serves of a preworkout supplement (217.5 mg per serve [73]). The average caffeine content of these products highlights the ease of accumulating a daily caffeine intake of this amount, though it should be noted that there is large variability between consumer products [39, 73]. Furthermore, the means of caffeine administration can alter the absorption rate and may impact the subsequent effect on sleep [74]. Importantly, consumers are unable to perceive sleep disturbance when consumption occurs further away from bedtime (i.e. 8 and 12 hours). Given the prevalence of high caffeine intake within 12 hours of bedtime, particularly among populations such as athletes [75, 76], shift workers [77, 78], armed forces personnel [79, 80], and students [81–83], the findings emphasize the need to raise awareness of the negative impact that a high dose (400 mg) of caffeine can have on subsequent sleep, even when consumed in the morning.

## Supplementary material

Supplementary material is available at *SLEEP* online.

zsae230_suppl_Supplementary_Tables_S1-S5_Figures_S1-S2

## Data Availability

The data underlying this article will be shared on reasonable request to the corresponding author.
