# Supplementary material for: Dose and timing effects of caffeine on subsequent sleep: a randomized clinical crossover trial
Source: Sleep. 2024 Oct 8;48(4):zsae230. doi: 10.1093/sleep/zsae230 (PMC11985402; doi:10.1093/sleep/zsae230)
Supplement: zsae230_suppl_Supplementary_Tables_S1-S5_Figures_S1-S2 [file zsae230_suppl_supplementary_tables_s1-s5_figures_s1-s2.zip › Supplementary Files.docx]

**SUPPLEMENTARY MATERIAL**

**Dose and timing effects of caffeine on subsequent sleep: A randomised clinical crossover trial**

Carissa L. Gardiner^1,2^, Jonathon Weakley^1,2,3*^, Louise M. Burke^4^, Francesca Fernandez^1^, Rich D. Johnston^1,2,3^, Josh Leota^5,6^, Suzanna Russell^1,2^, Gabriella Munteanu^1,2^, Andrew Townshend^1,2^, Shona L. Halson^1,2^

1. School of Behavioural and Health Sciences, Australian Catholic University, Brisbane, Australia
2. Sports Performance, Recovery, Injury and New Technologies (SPRINT) Research Centre, Australian Catholic University, Brisbane, Australia
3. Carnegie Applied Rugby Research (CARR) Centre, Institute of Sport, Physical Activity and Leisure, Leeds Beckett University, Leeds, UK
4. Exercise and Nutrition Research Program, Mary MacKillop Institute for Health Research, Australian Catholic University, Melbourne, Australia
5. School of Psychological Sciences, Monash University, Melbourne, Australia
6. Turner Institute for Brain and Mental Health, Monash University, Melbourne Australia

**Corresponding author: Carissa Gardiner**

Email: Carissa.Gardiner@acu.edu.au

School of Behavioural and Health Sciences

Australian Catholic University

Brisbane, Australia

**Supplementary Figure S1.** CONSORT flow diagram outlining the number of participants at each stage of the protocol.

**Supplementary Figure S2.** Density plots displaying total sleep time (minutes) for A) individuals carrying allele C (i.e., CC/CT genotype) and individuals with TT genotype for rs5751876 in *ADORA2A*; and B) individuals carrying allele C (i.e., CC/CA genotype) and individuals with AA genotype for rs762551 in *CYP1A2.*****

**Supplementary Table S1.** The Hardy–Weinberg equilibrium *p*-values and distribution of genotype frequencies of selected single nucleotide polymorphisms

| **Gene** | **Chromosomic location** | **SNP** | **Allele** | **MAF, %** | **HWE, *p*** |
| --- | --- | --- | --- | --- | --- |
| *ADORA2A* | 22 | rs5751876 | T>C | 57.8 | 0.31 |
| *CYP1A2* | 15 | rs762551 | C>A | 31.8 | 0.26 |

**Supplementary Table S2.** Comparisons for salivary caffeine concentrations

|  | **Estimate** | **Std error** | **df** | **t-ratio** | **p-value** | **Cohen's *d* (adj. 95% CI)** |
| --- | --- | --- | --- | --- | --- | --- |
| **PLACEBO CONTRASTS** | | | | | | |
| **400 mg; 4 h v placebo** | | | | | | |
| 11 h pre bed | -0.10 | 1.00 | 128.19 | -0.10 | 1.000 | -0.01 (-0.22 to 0.20); *trivial* |
| 7 h pre bed | 0.21 | 1.17 | 121.66 | 0.18 | 1.000 | 0.02 (-0.20 to 0.23); *trivial* |
| 3 h pre bed | 10.29 | 1.11 | 125.53 | 9.24 | <0.001* | 0.83 (0.58 to 1.07); *large* |
| 5 min pre bed | 10.21 | 0.90 | 124.11 | 11.31 | <0.001* | 1.02 (0.75 to 1.28); *large* |
| 5 min post wake | 3.02 | 0.43 | 125.73 | 7.02 | <0.001* | 0.63 (0.39 to 0.86); *medium* |
| **400 mg; 8 h v placebo** | | | | | | |
| 11 h pre bed | -0.16 | 0.99 | 128.00 | -0.16 | 1.000 | -0.01 (-0.23 to 0.20); *trivial* |
| 7 h pre bed | 11.31 | 1.14 | 121.55 | 9.95 | <0.001* | 0.90 (0.64 to 1.16); *large* |
| 3 h pre bed | 9.78 | 1.13 | 125.81 | 8.68 | <0.001* | 0.77 (0.53 to 1.02); *medium* |
| 5 min pre bed | 6.07 | 0.89 | 123.85 | 6.81 | <0.001* | 0.61 (0.38 to 0.85); *medium* |
| 5 min post wake | 2.41 | 0.43 | 125.22 | 5.63 | <0.001* | 0.50 (0.28 to 0.73); *medium* |
| **400 mg; 12 h v placebo** | | | | | | |
| 11 h pre bed | 9.71 | 0.99 | 128.00 | 9.86 | <0.001* | 0.87 (0.62 to 1.12); *large* |
| 7 h pre bed | 8.99 | 1.15 | 121.87 | 7.80 | <0.001* | 0.71 (0.46 to 0.95); *medium* |
| 3 h pre bed | 7.90 | 1.13 | 125.81 | 7.01 | <0.001* | 0.63 (0.39 to 0.86); *medium* |
| 5 min pre bed | 4.05 | 0.91 | 123.73 | 4.44 | <0.001* | 0.40 (0.18 to 0.62); *small* |
| 5 min post wake | 2.27 | 0.43 | 125.73 | 5.29 | <0.001* | 0.47 (0.25 to 0.70); *small* |
| **100 mg; 4 h v placebo** | | | | | | |
| 11 h pre bed | 0.75 | 0.99 | 128.00 | 0.76 | 1.000 | 0.07 (-0.15 to 0.28); *trivial* |
| 7 h pre bed | -0.08 | 1.17 | 122.15 | -0.07 | 1.000 | -0.01 (-0.22 to 0.21); *trivial* |
| 3 h pre bed | 4.87 | 1.11 | 125.53 | 4.37 | <0.001* | 0.39 (0.17 to 0.61); *small* |
| 5 min pre bed | 2.53 | 0.92 | 123.99 | 2.74 | 0.021* | 0.25 (0.03 to 0.46); *small* |
| 5 min post wake | 1.39 | 0.42 | 125.28 | 3.33 | 0.003* | 0.30 (0.08 to 0.52); *small* |
| **100 mg; 8 h v placebo** | | | | | | |
| 11 h pre bed | 0.40 | 1.01 | 128.39 | 0.40 | 1.000 | 0.04 (-0.18 to 0.25); *trivial* |
| 7 h pre bed | 4.85 | 1.12 | 121.28 | 4.32 | <0.001* | 0.39 (0.17 to 0.62); *small* |
| 3 h pre bed | 2.83 | 1.14 | 126.10 | 2.48 | 0.044* | 0.22 (0.00 to 0.44); *small* |
| 5 min pre bed | 2.30 | 0.89 | 123.85 | 2.58 | 0.033* | 0.23 (0.01 to 0.45); *small* |
| 5 min post wake | 0.72 | 0.42 | 125.28 | 1.73 | 0.259 | 0.15 (-0.06 to 0.37); *trivial* |
| **100 mg; 12 h v placebo** | | | | | | |
| 11 h pre bed | 3.04 | 1.00 | 128.19 | 3.05 | 0.008* | 0.27 (0.05 to 0.48); *small* |
| 7 h pre bed | 3.35 | 1.14 | 121.56 | 2.95 | 0.011* | 0.27 (0.05 to 0.49); *small* |
| 3 h pre bed | 1.87 | 1.13 | 125.81 | 1.66 | 0.300 | 0.15 (-0.07 to 0.36); *trivial* |
| 5 min pre bed | 0.82 | 0.89 | 123.85 | 0.92 | 1.000 | 0.08 (-0.13 to 0.30); *trivial* |
| 5 min post wake | 0.26 | 0.42 | 125.28 | 0.63 | 1.000 | 0.06 (-0.16 to 0.27); *trivial* |
| **DOSE CONTRASTS** | | | | | | |
| **400 mg; 4 h v 100 mg; 4 h** | | | | | | |
| 11 h pre bed | -0.84 | 1.00 | 128.19 | -0.84 | 1.000 | -0.07 (-0.29 to 0.14); *trivial* |
| 7 h pre bed | 0.29 | 1.20 | 122.12 | 0.24 | 1.000 | 0.02 (-0.20 to 0.24); *trivial* |
| 3 h pre bed | 5.42 | 1.08 | 125.01 | 5.00 | <0.001* | 0.45 (0.22 to 0.67); *small* |
| 5 min pre bed | 7.68 | 0.90 | 124.11 | 8.51 | <0.001* | 0.76 (0.52 to 1.01); *medium* |
| 5 min post wake | 1.62 | 0.42 | 125.43 | 3.83 | 0.001* | 0.34 (0.12 to 0.56); *small* |
| **400 mg; 8 h v 100 mg; 8 h** | | | | | | |
| 11 h pre bed | -0.56 | 1.01 | 128.39 | -0.56 | 1.000 | -0.05 (-0.26 to 0.16); *trivial* |
| 7 h pre bed | 6.46 | 1.12 | 121.28 | 5.76 | <0.001* | 0.52 (0.29 to 0.75); *medium* |
| 3 h pre bed | 6.95 | 1.13 | 125.81 | 6.17 | <0.001* | 0.55 (0.32 to 0.78); *medium* |
| 5 min pre bed | 3.77 | 0.86 | 123.02 | 4.41 | <0.001* | 0.40 (0.17 to 0.62); *small* |
| 5 min post wake | 1.69 | 0.42 | 125.49 | 3.97 | <0.001* | 0.36 (0.13 to 0.57); *small* |
| **400 mg; 12 h v 100 mg; 12 h** | | | | | | |
| 11 h pre bed | 6.68 | 1.00 | 128.19 | 6.69 | <0.001* | 0.59 (0.36 to 0.82); *medium* |
| 7 h pre bed | 5.64 | 1.15 | 121.87 | 4.89 | <0.001* | 0.44 (0.22 to 0.67); *small* |
| 3 h pre bed | 6.04 | 1.11 | 125.53 | 5.43 | <0.001* | 0.49 (0.26 to 0.71); *small* |
| 5 min pre bed | 3.23 | 0.88 | 123.62 | 3.68 | 0.001* | 0.33 (0.11 to 0.55); *small* |
| 5 min post wake | 2.01 | 0.42 | 125.43 | 4.74 | <0.001* | 0.42 (0.20 to 0.65); *small* |
| **TIMING CONTRASTS** | | | | | | |
| **400 mg; 4 h v 400 mg; 8h** | | | | | | |
| 11 h pre bed | 0.06 | 1.00 | 128.19 | 0.06 | 1.000 | 0.01 (-0.21 to 0.22); *trivial* |
| 7 h pre bed | -11.10 | 1.17 | 121.66 | -9.53 | <0.001* | -0.86 (-1.12 to -0.61); *large* |
| 3 h pre bed | 0.51 | 1.10 | 125.26 | 0.46 | 1.000 | 0.04 (-0.17 to 0.26); *trivial* |
| 5 min pre bed | 4.14 | 0.87 | 123.24 | 4.78 | <0.001* | 0.43 (0.20 to 0.66); *small* |
| 5 min post wake | 0.61 | 0.44 | 125.96 | 1.39 | 0.497 | 0.12 (-0.09 to 0.34); *trivial* |
| **400 mg; 4 h v 400 mg; 12h** | | | | | | |
| 11 h pre bed | -9.81 | 1.00 | 128.19 | -9.83 | <0.001* | -0.87 (-1.12 to -0.62); *large* |
| 7 h pre bed | -8.78 | 1.18 | 121.80 | -7.44 | <0.001* | -0.68 (-0.91 to -0.43); *medium* |
| 3 h pre bed | 2.38 | 1.10 | 125.26 | 2.17 | 0.096 | 0.19 (-0.02 to 0.41); *trivial* |
| 5 min pre bed | 6.16 | 0.89 | 123.87 | 6.93 | <0.001* | 0.62 (0.39 to 0.86); *medium* |
| 5 min post wake | 0.74 | 0.44 | 125.89 | 1.71 | 0.269 | 0.15 (-0.06 to 0.37); *trivial* |
| **400 mg; 8 h v 400 mg; 12h** | | | | | | |
| 11 h pre bed | -9.87 | 0.99 | 128.00 | -10.02 | <0.001* | -0.89 (-1.34 to -0.64); *large* |
| 7 h pre bed | 2.32 | 1.15 | 121.87 | 2.01 | 0.139 | 0.18 (-0.04 to 0.40); *trivial* |
| 3 h pre bed | 1.87 | 1.11 | 125.53 | 1.69 | 0.283 | 0.15 (-0.07 to 0.37); *trivial* |
| 5 min pre bed | 2.03 | 0.88 | 123.62 | 2.31 | 0.068 | 0.21 (-0.01 to 0.43); *small* |
| 5 min post wake | 0.14 | 0.44 | 125.96 | 0.32 | 1.000 | 0.03 (-0.19 to 0.24); *trivial* |
| **100 mg; 4 h v 100 mg; 8h** | | | | | | |
| 11 h pre bed | 0.34 | 1.01 | 128.39 | 0.34 | 1.000 | 0.03 (-0.18 to 0.24); *trivial* |
| 7 h pre bed | -4.93 | 1.16 | 121.83 | -4.27 | <0.001* | -0.39 (-0.61 to -0.16); *small* |
| 3 h pre bed | 2.04 | 1.11 | 125.53 | 1.83 | 0.207 | 0.16 (-0.05 to 0.38); *trivial* |
| 5 min pre bed | 0.23 | 0.89 | 123.85 | 0.26 | 1.000 | 0.02 (-0.19 to 0.24); *trivial* |
| 5 min post wake | 0.67 | 0.41 | 125.01 | 1.62 | 0.323 | 0.15 (-0.07 to 0.36); *trivial* |
| **100 mg; 4 h v 100 mg; 12h** | | | | | | |
| 11 h pre bed | -2.29 | 1.00 | 128.19 | -2.30 | 0.069 | -0.20 (-0.42 to 0.01); *small* |
| 7 h pre bed | -3.44 | 1.17 | 121.60 | -2.95 | 0.011* | -0.27 (-0.49 to -0.05); *small* |
| 3 h pre bed | 3.00 | 1.10 | 125.26 | 2.73 | 0.022* | 0.24 (0.03 to 0.46); *small* |
| 5 min pre bed | 1.72 | 0.89 | 123.85 | 1.93 | 0.170 | 0.17 (-0.04 to 0.39); *trivial* |
| 5 min post wake | 1.13 | 0.41 | 125.01 | 2.74 | 0.021* | 0.25 (0.03 to 0.46); *small* |
| **100 mg; 8 h v 100 mg; 12h** | | | | | | |
| 11 h pre bed | -2.63 | 1.02 | 128.60 | -2.57 | 0.034* | -0.23 (-0.44 to -0.01); *small* |
| 7 h pre bed | 1.49 | 1.12 | 121.28 | 1.33 | 0.557 | 0.12 (-0.10 to 0.34); *trivial* |
| 3 h pre bed | 0.96 | 1.13 | 125.81 | 0.85 | 1.000 | 0.08 (-0.14 to 0.29); *trivial* |
| 5 min pre bed | 1.48 | 0.86 | 123.02 | 1.74 | 0.256 | 0.16 (-0.06 to 0.37); *trivial* |
| 5 min post wake | 0.46 | 0.41 | 125.01 | 1.12 | 0.797 | 0.10 (-0.12 to 0.31); *trivial* |
| Abbreviations: CI– confidence interval; df- degrees of freedom  Notes: * p < 0.05; 95% CI adjusted for multiple comparisons | | | | | | |

**Supplementary Table S3.** Objective and subjective sleep outcomes for each condition (mean ± SD)

| **Outcome** | **Placebo** | **100 mg** | | | **400 mg** | | |
| --- | --- | --- | --- | --- | --- | --- | --- |
|  |  | **12 h** | **8 h** | **4 h** | **12 h** | **8 h** | **4 h** |
| **Objective sleep** | | | | | | | |
| Bedtime  (hh:mm ± min) | 22:51 ± 75.01 | 22:46 ± 73.48 | 23:02 ± 78.92 | 22:59 ± 76.30 | 23:00 ± 72.28 | 22:49 ± 65.89 | 22:56 ± 76.49 |
| Waketime  (hh:mm ± min) | 06:23 ± 62.68 | 06:19 ± 67.18 | 06:38 ± 67.79 | 06:32 ± 70.40 | 06:20 ± 66.90 | 06:23 ± 61.57 | 06:21 ± 55.99 |
| TIB  (min) | 446.89 ± 58.49 | 446.77 ± 49.17 | 449.28 ± 66.30 | 449.29 ± 72.94 | 433.83 ± 54.21 | 447.88 ± 52.82 | 440.14 ± 70.61 |
| TST  (min) | 406.96 ± 61.17 | 400.61 ± 44.45 | 398.98 ± 56.24 | 401.72 ± 59.27 | 378.71 ± 50.81 | 375.95 ± 55.91 | 358.10 ± 73.80 |
| SE  (%) | 91.17 ± 5.36 | 89.83 ± 6.24 | 88.70 ± 5.76 | 88.74 ± 6.28 | 87.16 ± 5.96 | 84.16 ± 10.13 | 81.58 ± 11.92 |
| SOL  (min) | 12.50 ± 16.76 | 14.07 ± 14.01 | 13.45 ± 18.91 | 16.60 ± 15.24 | 20.43 ± 23.43 | 15.64 ± 16.16 | 27.31 ± 34.29 |
| LPS  (min) | 18.52 ± 17.67 | 22.59 ± 17.31 | 21.52 ± 17.79 | 26.58 ± 21.09 | 33.80 ± 23.82 | 29.41 ± 23.67 | 45.33 ± 44.67 |
| ROL  (min) | 86.76 ± 20.99 | 80.84 ± 29.12 | 96.86 ± 43.16 | 78.42 ± 39.71 | 92.61 ± 60.97 | 89.10 ± 43.20 | 88.43 ± 48.26 |
| WASO  (min) | 29.95 ± 19.68 | 35.57 ± 28.66 | 41.12 ± 32.74 | 40.10 ± 27.59 | 38.62 ± 21.50 | 59.36 ± 49.49 | 56.45 ± 42.42 |
| Awakenings/h  (count) | 3.19 ±  1.39 | 2.92 ±  1.19 | 3.58 ±  1.90 | 3.62 ±  1.72 | 3.59 ±  1.73 | 4.03 ±  2.37 | 4.59 ±  1.99 |
| N1 & N2  (min) | 189.96 ± 33.69 | 193.59 ± 29.39 | 197.05 ± 36.99 | 188.65 ± 38.47 | 199.98 ± 33.24 | 191.48 ± 34.17 | 188.17 ± 41.48 |
| N1 & N2  (%) | 46.83 ± 6.11 | 48.57 ± 6.77 | 50.18 ± 11.49 | 46.96 ± 6.34 | 53.17 ± 7.68 | 51.22 ± 7.19 | 52.95 ± 9.22 |
| N3  (min) | 98.73 ± 30.92 | 96.11 ± 32.43 | 89.67 ± 36.56 | 104.40 ± 37.64 | 76.26 ± 36.93 | 82.40 ± 37.02 | 67.24 ± 29.60 |
| N3  (%) | 24.31 ± 6.57 | 24.11 ± 7.99 | 22.42 ± 8.55 | 26.57 ± 9.88 | 20.22 ± 9.53 | 21.80 ± 8.77 | 19.00 ± 8.04* |
| REM  (min) | 118.27 ± 32.52 | 110.91 ± 35.07 | 112.26 ± 37.51 | 108.67 ± 38.87 | 102.48 ± 33.77 | 102.07 ± 31.34 | 102.69 ± 38.55 |
| REM  (%) | 28.84 ± 5.53 | 27.30 ± 7.70 | 27.41 ± 7.88 | 26.50 ± 7.21 | 26.62 ± 6.80 | 26.98 ± 6.43 | 28.07 ± 6.73 |
| **Subjective sleep** | | | | | | | |
| TST  (h) | 7.48 ± 0.91 | 6.84 ± 0.99 | 7.33 ± 1.16 | 7.14 ± 0.85 | 6.91 ± 1.00 | 6.79 ± 1.03 | 6.20 ± 1.44 |
| SOL  (min) | 11.23 ± 7.09 | 18.36 ± 17.91 | 13.67 ± 12.56 | 18.48 ± 13.75 | 23.13 ± 20.83 | 22.95 ± 16.42 | 43.81 ± 37.94 |
| WASO  (min) | 15.18 ± 21.81 | 18.14 ± 21.36 | 13.43 ± 14.61 | 10.52 ± 10.85 | 19.65 ± 15.84 | 21.91 ± 30.27 | 28.91 ± 44.96 |
| Sleep quality  (units) | 3.44 ± 0.73 | 3.18 ± 0.91 | 3.14 ± 0.99 | 3.19 ± 0.60 | 3.00 ± 0.95 | 2.96 ± 1.17 | 2.57 ± 1.03 |
| KSS  (units) | 6.68 ± 1.29 | 5.82 ± 1.40 | 6.30 ± 1.66 | 6.23 ± 1.41 | 5.96 ± 1.76 | 5.91 ± 2.06 | 5.48 ± 2.06 |
| Presleep alertness  (units) | 2.43 ±0.66 | 2.59 ± 0.67 | 2.68 ± 0.65 | 2.50 ± 0.51 | 2.65 ± 0.71 | 2.68 ± 0.78 | 3.01 ± 0.85 |
| Abbreviations: KSS- Karolinska Sleepiness Scale; LPS- sleep onset latency to persistent sleep; N1- non-rapid eye movement (NREM) stage 1 sleep; N2- NREM stage 2 sleep; N3- NREM stage 3 sleep; REM- rapid eye movement (REM) sleep; ROL- REM sleep onset latency; SE- sleep efficiency; SOL- sleep onset latency; TIB- time in bed; TST- total sleep time; WASO- wake after sleep onset | | | | | | | |

**Supplementary Table S4.** Comparisons for objective sleep outcomes

|  | **Estimate** | **Std error** | **df** | **t-ratio** | **p-value** | **Cohen's *d* (adj. 95% CI)** |
| --- | --- | --- | --- | --- | --- | --- |
| **PLACEBO CONTRASTS** | | | | | | |
| **400 mg; 4 h v placebo** | | | | | | |
| TST (min) | -50.64 | 12.73 | 119.78 | -3.98 | <0.001* | -0.36 (-0.59 to -0.14); *small* |
| SE (%) | -9.53 | 1.81 | 119.97 | -5.27 | <0.001* | -0.48 (-0.71 to -0.25); *small* |
| SOL (min) | 14.16 | 4.87 | 123.20 | 2.91 | 0.013* | 0.26 (0.04 to 0.48); *small* |
| LPS (min) | 25.39 | 5.62 | 123.17 | 4.52 | <0.001* | 0.41 (0.18 to 0.63); *small* |
| ROL (min) | -1.31 | 11.17 | 122.44 | -0.12 | 1.000 | -0.01 (-0.23 to 0.21); *trivial* |
| WASO (min) | 26.18 | 7.56 | 119.89 | 3.46 | 0.002* | 0.32 (0.09 to 0.54); *small* |
| Awakenings/h (count) | 1.40 | 0.39 | 119.78 | 3.59 | 0.001* | 0.33 (0.10 to 0.55); *small* |
| N1 & N2 (min) | -4.46 | 8.60 | 120.02 | -0.52 | 1.000 | -0.05 (-0.27 to 0.17); *trivial* |
| N1 & N2 (%) | 5.58 | 1.79 | 119.81 | 3.11 | 0.007* | 0.28 (0.06 to 0.51); *small* |
| N3 (min) | -29.65 | 5.42 | 119.32 | -5.47 | <0.001* | -0.50 (-0.73 to -0.27); *medium* |
| N3 (%) | -4.74 | 1.47 | 119.40 | -3.24 | 0.005* | -0.30 (-0.52 to -0.07); *small* |
| REM (min) | -16.06 | 7.27 | 119.66 | -2.21 | 0.087 | -0.20 (-0.42 to 0.02); *small* |
| REM (%) | -0.71 | 1.49 | 119.75 | -0.48 | 1.000 | -0.04 (-0.26 to 0.18); *trivial* |
| **400 mg; 8 h v placebo** | | | | | | |
| TST (min) | -28.66 | 12.66 | 119.16 | -2.26 | 0.076 | -0.21 (-0.43 to 0.02); *small* |
| SE (%) | -6.88 | 1.80 | 119.21 | -3.82 | 0.001* | -0.35 (-0.58 to -0.12); *small* |
| SOL (min) | 3.47 | 4.94 | 123.46 | 0.70 | 1.000 | 0.06 (-0.15 to 0.28); *trivial* |
| LPS (min) | 11.64 | 5.70 | 123.39 | 2.04 | 0.129 | 0.18 (-0.03 to 0.40); *trivial* |
| ROL (min) | 1.75 | 11.32 | 122.78 | 0.16 | 1.000 | 0.01 (-0.20 to 0.23); *trivial* |
| WASO (min) | 29.11 | 7.52 | 119.19 | 3.87 | 0.001* | 0.36 (0.13 to 0.58); *small* |
| Awakenings/h (count) | 0.86 | 0.39 | 119.16 | 2.22 | 0.086 | 0.20 (-0.02 to 0.42); *small* |
| N1 & N2 (min) | 2.04 | 8.55 | 119.22 | 0.24 | 1.000 | 0.02 (-0.20 to 0.24); *trivial* |
| N1 & N2 (%) | 4.24 | 1.78 | 119.17 | 2.38 | 0.056 | 0.22 (0.00 to 0.44); *small* |
| N3 (min) | -15.29 | 5.39 | 119.06 | -2.84 | 0.016* | -0.26 (-0.48 to -0.04); *small* |
| N3 (%) | -2.41 | 1.46 | 119.07 | -1.66 | 0.301 | -0.15 (-0.37 to 0.07); *trivial* |
| REM (min) | -15.32 | 7.23 | 119.13 | -2.12 | 0.109 | -0.19 (-0.42 to 0.03); *trivial* |
| REM (%) | -1.80 | 1.48 | 119.15 | -1.22 | 0.680 | -0.11 (-0.33 to 0.11); *trivial* |
| **400 mg; 12 h v placebo** | | | | | | |
| TST (min) | -30.02 | 12.73 | 119.78 | -2.36 | 0.060 | -0.22 (-0.44 to 0.01); *small* |
| SE (%) | -3.94 | 1.81 | 119.97 | -2.18 | 0.094 | -0.20 (-0.42 to 0.02); *small* |
| SOL (min) | 7.94 | 4.81 | 123.01 | 1.65 | 0.305 | 0.15 (-0.07 to 0.37); *trivial* |
| LPS (min) | 15.28 | 5.55 | 123.01 | 2.76 | 0.020* | 0.25 (0.03 to 0.47); *small* |
| ROL (min) | 5.51 | 11.17 | 122.44 | 0.49 | 1.000 | 0.05 (-0.17 to 0.26); *trivial* |
| WASO (min) | 8.35 | 7.56 | 119.89 | 1.10 | 0.815 | 0.10 (-0.12 to 0.32); *trivial* |
| Awakenings/h (count) | 0.40 | 0.39 | 119.78 | 1.02 | 0.937 | 0.09 (-0.13 to 0.31); *trivial* |
| N1 & N2 (min) | 7.35 | 8.60 | 120.02 | 0.86 | 1.000 | 0.08 (-0.14 to 0.30); *trivial* |
| N1 & N2 (%) | 5.79 | 1.79 | 119.81 | 3.23 | 0.005* | 0.30 (0.07 to 0.52); *small* |
| N3 (min) | -20.63 | 5.42 | 119.32 | -3.80 | 0.001* | -0.35 (-0.57 to -0.12); *small* |
| N3 (%) | -3.52 | 1.47 | 119.40 | -2.40 | 0.054 | -0.22 (-0.44 to 0.00); *small* |
| REM (min) | -16.27 | 7.27 | 119.66 | -2.24 | 0.081 | -0.21 (-0.43 to 0.02); *small* |
| REM (%) | -2.17 | 1.49 | 119.75 | -1.46 | 0.444 | -0.13 (-0.35 to 0.09); *trivial* |
| **100 mg; 4 h v placebo** | | | | | | |
| TST (min) | -4.47 | 12.85 | 119.35 | -0.35 | 1.000 | -0.03 (-0.25 to 0.19); *trivial* |
| SE (%) | -2.44 | 1.83 | 119.45 | -1.33 | 0.554 | -0.12 (-0.34 to 0.10); *trivial* |
| SOL (min) | 3.94 | 5.01 | 123.73 | 0.79 | 1.000 | 0.07 (-0.15 to 0.29); *trivial* |
| LPS (min) | 7.94 | 5.78 | 123.61 | 1.37 | 0.516 | 0.12 (-0.09 to 0.34); *trivial* |
| ROL (min) | -9.04 | 11.49 | 123.20 | -0.79 | 1.000 | -0.07 (-0.29 to 0.15); *trivial* |
| WASO (min) | 10.92 | 7.63 | 119.41 | 1.43 | 0.465 | 0.13 (-0.09 to 0.35); *trivial* |
| Awakenings/h (count) | 0.50 | 0.39 | 119.35 | 1.29 | 0.604 | 0.12 (-0.10 to 0.34); *trivial* |
| N1 & N2 (min) | -0.68 | 8.68 | 119.48 | -0.08 | 1.000 | -0.01 (-0.23 to 0.21); *trivial* |
| N1 & N2 (%) | 0.19 | 1.81 | 119.37 | 0.11 | 1.000 | 0.01 (-0.21 to 0.23); *trivial* |
| N3 (min) | 4.19 | 5.47 | 119.13 | 0.77 | 1.000 | 0.07 (-0.15 to 0.29); *trivial* |
| N3 (%) | 1.86 | 1.48 | 119.17 | 1.26 | 0.636 | 0.12 (-0.11 to 0.34); *trivial* |
| REM (min) | -8.17 | 7.34 | 119.29 | -1.11 | 0.804 | -0.10 (-0.32 to 0.12); *trivial* |
| REM (%) | -2.03 | 1.50 | 119.33 | -1.35 | 0.537 | -0.12 (-0.34 to 0.10); *trivial* |
| **100 mg; 8 h v placebo** | | | | | | |
| TST (min) | -7.58 | 12.73 | 119.76 | -0.60 | 1.000 | -0.05 (-0.27 to 0.17); *trivial* |
| SE (%) | -2.17 | 1.81 | 119.95 | -1.20 | 0.698 | -0.11 (-0.33 to 0.11); *trivial* |
| SOL (min) | 0.67 | 4.94 | 123.46 | 0.14 | 1.000 | 0.01 (-0.20 to 0.23); *trivial* |
| LPS (min) | 2.63 | 5.70 | 123.39 | 0.46 | 1.000 | 0.04 (-0.17 to 0.26); *trivial* |
| ROL (min) | 8.47 | 11.32 | 122.78 | 0.75 | 1.000 | 0.07 (-0.15 to 0.28); *trivial* |
| WASO (min) | 10.10 | 7.56 | 119.87 | 1.34 | 0.552 | 0.12 (-0.10 to 0.34); *trivial* |
| Awakenings/h (count) | 0.38 | 0.39 | 119.76 | 0.97 | 1.000 | 0.09 (-0.13 to 0.31); *trivial* |
| N1 & N2 (min) | 7.02 | 8.59 | 120.00 | 0.82 | 1.000 | 0.08 (-0.14 to 0.29); *trivial* |
| N1 & N2 (%) | 3.31 | 1.79 | 119.80 | 1.85 | 0.201 | 0.17 (-0.05 to 0.39); *trivial* |
| N3 (min) | -8.39 | 5.42 | 119.32 | -1.55 | 0.374 | -0.14 (-0.36 to 0.08); *trivial* |
| N3 (%) | -1.79 | 1.47 | 119.39 | -1.22 | 0.671 | -0.11 (-0.33 to 0.11); *trivial* |
| REM (min) | -6.08 | 7.27 | 119.64 | -0.84 | 1.000 | -0.08 (-0.30 to 0.14); *trivial* |
| REM (%) | -1.46 | 1.49 | 119.73 | -0.98 | 0.989 | -0.09 (-0.31 to 0.13); *trivial* |
| **100 mg; 12 h v placebo** | | | | | | |
| TST (min) | -7.20 | 12.54 | 119.42 | -0.57 | 1.000 | -0.05 (-0.27 to 0.17); *trivial* |
| SE (%) | -1.15 | 1.78 | 119.54 | -0.65 | 1.000 | -0.06 (-0.28 to 0.16); *trivial* |
| SOL (min) | 1.31 | 4.87 | 123.20 | 0.27 | 1.000 | 0.02 (-0.19 to 0.24); *trivial* |
| LPS (min) | 3.63 | 5.62 | 123.17 | 0.65 | 1.000 | 0.06 (-0.16 to 0.27); *trivial* |
| ROL (min) | -6.38 | 11.17 | 122.36 | -0.57 | 1.000 | -0.05 (-0.27 to 0.17); *trivial* |
| WASO (min) | 5.39 | 7.45 | 119.49 | 0.72 | 1.000 | 0.07 (-0.15 to 0.29); *trivial* |
| Awakenings/h (count) | -0.21 | 0.38 | 119.42 | -0.55 | 1.000 | -0.05 (-0.27 to 0.17); *trivial* |
| N1 & N2 (min) | 1.57 | 8.47 | 119.56 | 0.19 | 1.000 | 0.02 (-0.20 to 0.24); *trivial* |
| N1 & N2 (%) | 1.32 | 1.76 | 119.44 | 0.75 | 1.000 | 0.07 (-0.15 to 0.29); *trivial* |
| N3 (min) | -0.59 | 5.34 | 119.17 | -0.11 | 1.000 | -0.01 (-0.23 to 0.21); *trivial* |
| N3 (%) | 0.35 | 1.44 | 119.21 | 0.24 | 1.000 | 0.02 (-0.20 to 0.24); *trivial* |
| REM (min) | -7.76 | 7.16 | 119.36 | -1.08 | 0.843 | -0.10 (-0.32 to 0.12); *trivial* |
| REM (%) | -1.59 | 1.47 | 119.41 | -1.09 | 0.837 | -0.10 (-0.32 to 0.12); *trivial* |
| **DOSE CONTRASTS** | | | | | | |
| **400 mg; 4 h v 100 mg; 4 h** | | | | | | |
| TST (min) | -46.17 | 13.09 | 120.16 | -3.53 | 0.002* | -0.32 (-0.55 to -0.10); *small* |
| SE (%) | -7.09 | 1.86 | 120.46 | -3.81 | 0.001* | -0.35 (-0.57 to -0.12); *small* |
| SOL (min) | 10.23 | 5.08 | 123.96 | 2.02 | 0.138 | 0.18 (-0.04 to 0.40); *trivial* |
| LPS (min) | 17.45 | 5.85 | 123.81 | 2.98 | 0.010* | 0.27 (0.05 to 0.49); *small* |
| ROL (min) | 7.73 | 11.63 | 123.69 | 0.67 | 1.000 | 0.06 (-0.16 to 0.28); *trivial* |
| WASO (min) | 15.26 | 7.77 | 120.33 | 1.96 | 0.156 | 0.18 (-0.04 to 0.40); *trivial* |
| Awakenings/h (count) | 0.89 | 0.40 | 120.16 | 2.23 | 0.084 | 0.20 (-0.02 to 0.42); *small* |
| N1 & N2 (min) | -3.78 | 8.84 | 120.53 | -0.43 | 1.000 | -0.04 (-0.26 to 0.18); *trivial* |
| N1 & N2 (%) | 5.39 | 1.84 | 120.22 | 2.92 | 0.012* | 0.27 (0.04 to 0.49); *small* |
| N3 (min) | -33.84 | 5.58 | 119.48 | -6.06 | <0.001* | -0.56 (-0.79 to -0.32); *medium* |
| N3 (%) | -6.60 | 1.51 | 119.60 | -4.38 | <0.001* | -0.40 (-0.63 to -0.17); *small* |
| REM (min) | -7.89 | 7.48 | 119.98 | -1.06 | 0.881 | -0.10 (-0.32 to 0.12); *trivial* |
| REM (%) | 1.32 | 1.53 | 120.12 | 0.86 | 1.000 | 0.08 (-0.14 to 0.30); *trivial* |
| **400 mg; 8 h v 100 mg; 8 h** | | | | | | |
| TST (min) | -21.07 | 12.90 | 119.93 | -1.63 | 0.315 | -0.15 (-0.37 to 0.07); *trivial* |
| SE (%) | -4.71 | 1.83 | 120.16 | -2.57 | 0.034* | -0.23 (-0.46 to -0.01); *small* |
| SOL (min) | 2.80 | 5.07 | 123.98 | 0.55 | 1.000 | 0.05 (-0.17 to 0.27); *trivial* |
| LPS (min) | 9.01 | 5.85 | 123.82 | 1.54 | 0.378 | 0.14 (-0.08 to 0.35); *trivial* |
| ROL (min) | -6.72 | 11.61 | 123.60 | -0.58 | 1.000 | -0.05 (-0.27 to 0.16); *trivial* |
| WASO (min) | 19.01 | 7.66 | 120.06 | 2.48 | 0.043* | 0.23 (0.01 to 0.45); *small* |
| Awakenings/h (count) | 0.48 | 0.39 | 119.92 | 1.22 | 0.676 | 0.11 (-0.11 to 0.33); *trivial* |
| N1 & N2 (min) | -4.98 | 8.71 | 120.22 | -0.57 | 1.000 | -0.05 (-0.27 to 0.17); *trivial* |
| N1 & N2 (%) | 0.93 | 1.81 | 119.97 | 0.52 | 1.000 | 0.05 (-0.17 to 0.27); *trivial* |
| N3 (min) | -6.90 | 5.50 | 119.38 | -1.26 | 0.635 | -0.12 (-0.33 to 0.11); *trivial* |
| N3 (%) | -0.62 | 1.49 | 119.48 | -0.42 | 1.000 | -0.04 (-0.26 to 0.18); *trivial* |
| REM (min) | -9.24 | 7.37 | 119.78 | -1.25 | 0.638 | -0.12 (-0.33 to 0.11); *trivial* |
| REM (%) | -0.34 | 1.51 | 119.89 | -0.23 | 1.000 | -0.02 (-0.24 to 0.20); *trivial* |
| **400 mg; 12 h v 100 mg; 12 h** | | | | | | |
| TST (min) | -22.82 | 12.72 | 119.62 | -1.79 | 0.226 | -0.16 (-0.38 to 0.06); *trivial* |
| SE (%) | -2.79 | 1.81 | 119.79 | -1.54 | 0.376 | -0.14 (-0.36 to 0.08); *trivial* |
| SOL (min) | 6.63 | 4.87 | 123.20 | 1.36 | 0.528 | 0.12 (-0.09 to 0.34); *trivial* |
| LPS (min) | 11.66 | 5.62 | 123.17 | 2.08 | 0.120 | 0.19 (-0.03 to 0.40); *trivial* |
| ROL (min) | 11.89 | 11.31 | 122.79 | 1.05 | 0.886 | 0.10 (-0.12 to 0.31); *trivial* |
| WASO (min) | 2.95 | 7.55 | 119.72 | 0.39 | 1.000 | 0.04 (-0.18 to 0.26); *trivial* |
| Awakenings/h (count) | 0.60 | 0.39 | 119.62 | 1.56 | 0.367 | 0.14 (-0.08 to 0.36); *trivial* |
| N1 & N2 (min) | 5.78 | 8.59 | 119.83 | 0.67 | 1.000 | 0.06 (-0.16 to 0.28); *trivial* |
| N1 & N2 (%) | 4.47 | 1.79 | 119.65 | 2.50 | 0.041* | 0.23 (0.01 to 0.45); *small* |
| N3 (min) | -20.04 | 5.42 | 119.25 | -3.70 | 0.001* | -0.34 (-0.56 to -0.11); *small* |
| N3 (%) | -3.87 | 1.46 | 119.31 | -2.64 | 0.028* | -0.24 (-0.46 to -0.02); *small* |
| REM (min) | -8.52 | 7.26 | 119.52 | -1.17 | 0.730 | -0.11 (-0.33 to 0.11); *trivial* |
| REM (%) | -0.57 | 1.49 | 119.60 | -0.39 | 1.000 | -0.04 (-0.25 to 0.18); *trivial* |
| **TIMING CONTRASTS** | | | | | | |
| **400 mg; 4 h v 400 mg; 8h** | | | | | | |
| TST (min) | -21.98 | 12.90 | 119.94 | -1.70 | 0.273 | -0.16 (-0.38 to 0.07); *trivial* |
| SE (%) | -2.65 | 1.83 | 120.18 | -1.44 | 0.454 | -0.13 (-0.35 to 0.09); *trivial* |
| SOL (min) | 10.69 | 5.00 | 123.68 | 2.14 | 0.104 | 0.19 (-0.03 to 0.41); *trivial* |
| LPS (min) | 13.75 | 5.77 | 123.57 | 2.38 | 0.056 | 0.22 (0.00 to 0.43); *small* |
| ROL (min) | -3.06 | 11.46 | 123.23 | -0.27 | 1.000 | -0.02 (-0.24 to 0.19); *trivial* |
| WASO (min) | -2.93 | 7.66 | 120.08 | -0.38 | 1.000 | -0.04 (-0.25 to 0.18); *trivial* |
| Awakenings/h (count) | 0.54 | 0.39 | 119.94 | 1.36 | 0.526 | 0.13 (-0.10 to 0.34); *trivial* |
| N1 & N2 (min) | -6.50 | 8.71 | 120.24 | -0.75 | 1.000 | -0.07 (-0.29 to 0.15); *trivial* |
| N1 & N2 (%) | 1.33 | 1.82 | 119.99 | 0.73 | 1.000 | 0.07 (-0.15 to 0.29); *trivial* |
| N3 (min) | -14.36 | 5.50 | 119.39 | -2.61 | 0.031* | -0.24 (-0.46 to -0.02); *small* |
| N3 (%) | -2.33 | 1.49 | 119.49 | -1.57 | 0.358 | -0.14 (-0.36 to 0.08); *trivial* |
| REM (min) | -0.74 | 7.37 | 119.80 | -0.10 | 1.000 | -0.01 (-0.23 to 0.21); *trivial* |
| REM (%) | 1.09 | 1.51 | 119.91 | 0.72 | 1.000 | 0.07 (-0.15 to 0.28); *trivial* |
| **400 mg; 4 h v 400 mg; 12h** | | | | | | |
| TST (min) | -20.62 | 12.79 | 119.02 | -1.61 | 0.328 | -0.15 (-0.37 to 0.07); *trivial* |
| SE (%) | -5.59 | 1.82 | 119.03 | -3.07 | 0.008* | -0.28 (-0.51 to -0.06); *small* |
| SOL (min) | 6.23 | 4.87 | 123.20 | 1.28 | 0.611 | 0.12 (-0.10 to 0.33); *trivial* |
| LPS (min) | 10.11 | 5.62 | 123.17 | 1.80 | 0.223 | 0.16 (-0.06 to 0.38); *trivial* |
| ROL (min) | -6.82 | 11.28 | 122.04 | -0.61 | 1.000 | -0.06 (-0.27 to 0.16); *trivial* |
| WASO (min) | 17.83 | 7.59 | 119.02 | 2.35 | 0.061 | 0.22 (-0.01 to 0.44); *small* |
| Awakenings/h (count) | 1.00 | 0.39 | 119.02 | 2.56 | 0.035* | 0.24 (0.01 to 0.46); *small* |
| N1 & N2 (min) | -11.81 | 8.64 | 119.03 | -1.37 | 0.522 | -0.13 (-0.35 to 0.10); *trivial* |
| N1 & N2 (%) | -0.21 | 1.80 | 119.02 | -0.12 | 1.000 | -0.01 (-0.23 to 0.21); *trivial* |
| N3 (min) | -9.02 | 5.44 | 119.00 | -1.66 | 0.300 | -0.15 (-0.37 to 0.07); *trivial* |
| N3 (%) | -1.22 | 1.47 | 119.01 | -0.83 | 1.000 | -0.08 (-0.30 to 0.14); *trivial* |
| REM (min) | 0.21 | 7.30 | 119.01 | 0.03 | 1.000 | 0.00 (-0.22 to 0.22); *trivial* |
| REM (%) | 1.45 | 1.49 | 119.02 | 0.97 | 0.998 | 0.09 (-0.13 to 0.31); *trivial* |
| **400 mg; 8 h v 400 mg; 12h** | | | | | | |
| TST (min) | 1.36 | 12.90 | 119.94 | 0.11 | 1.000 | 0.01 (-0.21 to 0.23); *trivial* |
| SE (%) | -2.94 | 1.83 | 120.18 | -1.60 | 0.335 | -0.15 (-0.37 to 0.07); *trivial* |
| SOL (min) | -4.46 | 4.94 | 123.46 | -0.90 | 1.000 | -0.08 (-0.30 to 0.14); *trivial* |
| LPS (min) | -3.65 | 5.70 | 123.39 | -0.64 | 1.000 | -0.06 (-0.27 to 0.16); *trivial* |
| ROL (min) | -3.76 | 11.46 | 123.23 | -0.33 | 1.000 | -0.03 (-0.25 to 0.19); *trivial* |
| WASO (min) | 20.76 | 7.66 | 120.08 | 2.71 | 0.023* | 0.25 (0.03 to 0.47); *small* |
| Awakenings/h (count) | 0.46 | 0.39 | 119.94 | 1.17 | 0.728 | 0.11 (-0.11 to 0.33); *trivial* |
| N1 & N2 (min) | -5.31 | 8.71 | 120.24 | -0.61 | 1.000 | -0.06 (-0.27 to 0.16); *trivial* |
| N1 & N2 (%) | -1.55 | 1.82 | 119.99 | -0.85 | 1.000 | -0.08 (-0.30 to 0.14); *trivial* |
| N3 (min) | 5.34 | 5.50 | 119.39 | 0.97 | 1.000 | 0.09 (-0.13 to 0.31); *trivial* |
| N3 (%) | 1.11 | 1.49 | 119.49 | 0.75 | 1.000 | 0.07 (-0.15 to 0.29); *trivial* |
| REM (min) | 0.95 | 7.37 | 119.80 | 0.13 | 1.000 | 0.01 (-0.21 to 0.23); *trivial* |
| REM (%) | 0.37 | 1.51 | 119.91 | 0.24 | 1.000 | 0.02 (-0.20 to 0.24); *trivial* |
| **100 mg; 4 h v 100 mg; 8h** | | | | | | |
| TST (min) | 3.11 | 13.04 | 119.75 | 0.24 | 1.000 | 0.02 (-0.20 to 0.24); *trivial* |
| SE (%) | -0.27 | 1.85 | 119.95 | -0.14 | 1.000 | -0.01 (-0.23 to 0.21); *trivial* |
| SOL (min) | 3.26 | 5.13 | 123.72 | 0.64 | 1.000 | 0.06 (-0.16 to 0.27); *trivial* |
| LPS (min) | 5.31 | 5.91 | 123.60 | 0.90 | 1.000 | 0.08 (-0.14 to 0.30); *trivial* |
| ROL (min) | -17.51 | 11.74 | 123.19 | -1.49 | 0.415 | -0.13 (-0.35 to 0.08); *trivial* |
| WASO (min) | 0.82 | 7.74 | 119.86 | 0.11 | 1.000 | 0.01 (-0.21 to 0.23); *trivial* |
| Awakenings/h (count) | 0.13 | 0.40 | 119.75 | 0.32 | 1.000 | 0.03 (-0.19 to 0.25); *trivial* |
| N1 & N2 (min) | -7.70 | 8.80 | 119.99 | -0.88 | 1.000 | -0.08 (-0.30 to 0.14); *trivial* |
| N1 & N2 (%) | -3.12 | 1.83 | 119.79 | -1.70 | 0.275 | -0.16 (-0.38 to 0.07); *trivial* |
| N3 (min) | 12.58 | 5.56 | 119.31 | 2.26 | 0.076 | 0.21 (-0.02 to 0.43); *small* |
| N3 (%) | 3.65 | 1.50 | 119.39 | 2.43 | 0.050 | 0.22 (0.00 to 0.44); *small* |
| REM (min) | -2.09 | 7.45 | 119.64 | -0.28 | 1.000 | -0.03 (-0.24 to 0.19); *trivial* |
| REM (%) | -0.57 | 1.52 | 119.73 | -0.38 | 1.000 | -0.03 (-0.25 to 0.19); *trivial* |
| **100 mg; 4 h v 100 mg; 12h** | | | | | | |
| TST (min) | 2.73 | 12.90 | 119.76 | 0.21 | 1.000 | 0.02 (-0.20 to 0.24); *trivial* |
| SE (%) | -1.29 | 1.83 | 119.97 | -0.70 | 1.000 | -0.06 (-0.28 to 0.16); *trivial* |
| SOL (min) | 2.63 | 5.08 | 123.96 | 0.52 | 1.000 | 0.05 (-0.17 to 0.26); *trivial* |
| LPS (min) | 4.32 | 5.85 | 123.81 | 0.74 | 1.000 | 0.07 (-0.15 to 0.28); *trivial* |
| ROL (min) | -2.66 | 11.63 | 123.58 | -0.23 | 1.000 | -0.02 (-0.24 to 0.20); *trivial* |
| WASO (min) | 5.53 | 7.66 | 119.88 | 0.72 | 1.000 | 0.07 (-0.15 to 0.29); *trivial* |
| Awakenings/h (count) | 0.71 | 0.39 | 119.76 | 1.81 | 0.218 | 0.17 (-0.06 to 0.39); *trivial* |
| N1 & N2 (min) | -2.24 | 8.71 | 120.02 | -0.26 | 1.000 | -0.02 (-0.24 to 0.20); *trivial* |
| N1 & N2 (%) | -1.13 | 1.82 | 119.80 | -0.62 | 1.000 | -0.06 (-0.28 to 0.16); *trivial* |
| N3 (min) | 4.78 | 5.50 | 119.30 | 0.87 | 1.000 | 0.08 (-0.14 to 0.30); *trivial* |
| N3 (%) | 1.51 | 1.49 | 119.38 | 1.02 | 0.937 | 0.09 (-0.13 to 0.31); *trivial* |
| REM (min) | -0.41 | 7.37 | 119.64 | -0.06 | 1.000 | -0.01 (-0.22 to 0.21); *trivial* |
| REM (%) | -0.44 | 1.51 | 119.73 | -0.29 | 1.000 | -0.03 (-0.25 to 0.19); *trivial* |
| **100 mg; 8 h v 100 mg; 12h** | | | | | | |
| TST (min) | -0.38 | 12.72 | 119.61 | -0.03 | 1.000 | 0.00 (-0.22 to 0.22); *trivial* |
| SE (%) | -1.02 | 1.81 | 119.78 | -0.56 | 1.000 | -0.05 (-0.27 to 0.17); *trivial* |
| SOL (min) | -0.63 | 5.00 | 123.68 | -0.13 | 1.000 | -0.01 (-0.23 to 0.20); *trivial* |
| LPS (min) | -1.00 | 5.77 | 123.57 | -0.17 | 1.000 | -0.02 (-0.23 to 0.20); *trivial* |
| ROL (min) | 14.85 | 11.46 | 123.14 | 1.30 | 0.592 | 0.12 (-0.10 to 0.33); *trivial* |
| WASO (min) | 4.71 | 7.55 | 119.71 | 0.62 | 1.000 | 0.06 (-0.16 to 0.28); *trivial* |
| Awakenings/h (count) | 0.59 | 0.39 | 119.61 | 1.51 | 0.400 | 0.14 (-0.08 to 0.36); *trivial* |
| N1 & N2 (min) | 5.46 | 8.59 | 119.81 | 0.64 | 1.000 | 0.06 (-0.16 to 0.28); *trivial* |
| N1 & N2 (%) | 1.99 | 1.79 | 119.64 | 1.11 | 0.804 | 0.10 (-0.12 to 0.32); *trivial* |
| N3 (min) | -7.80 | 5.42 | 119.25 | -1.44 | 0.458 | -0.13 (-0.35 to 0.09); *trivial* |
| N3 (%) | -2.14 | 1.46 | 119.31 | -1.46 | 0.439 | -0.13 (-0.35 to 0.09); *trivial* |
| REM (min) | 1.67 | 7.26 | 119.51 | 0.23 | 1.000 | 0.02 (-0.20 to 0.24); *trivial* |
| REM (%) | 0.14 | 1.49 | 119.59 | 0.09 | 1.000 | 0.01 (-0.21 to 0.23); *trivial* |
| Abbreviations: CI– confidence interval; df- degrees of freedom; KSS- Karolinska Sleepiness Scale; LPS- sleep onset latency to persistent sleep; N1- non-rapid eye movement (NREM) stage 1 sleep; N2- NREM stage 2 sleep; N3- NREM stage 3 sleep; REM- rapid eye movement (REM) sleep; ROL- REM sleep onset latency; SE- sleep efficiency; SOL- sleep onset latency; TIB- time in bed; TST- total sleep time; WASO- wake after sleep onset  Notes: * p < 0.05; 95% CI adjusted for multiple comparisons | | | | | | |

**Supplementary Table S5**. Comparisons for subjective sleep outcomes

|  | **Estimate** | **Std error** | **df** | **t-ratio** | **p-value** | **Cohen's *d* (adj. 95% CI)** |
| --- | --- | --- | --- | --- | --- | --- |
| **PLACEBO CONTRASTS** | | | | | | |
| **400 mg; 4 h v placebo** | | | | | | |
| Subjective TST (min) | -1.26 | 0.26 | 124.55 | -4.91 | <0.001* | -0.44 (-0.66 to -0.22); *small* |
| Subjective SOL (min) | 31.30 | 5.61 | 125.57 | 5.58 | <0.001* | 0.50 (0.27 to 0.72); *medium* |
| Subjective WASO (min) | 10.58 | 6.64 | 126.44 | 1.59 | 0.341 | 0.14 (-0.07 to 0.36); *trivial* |
| Subjective quality (units) | -0.83 | 0.26 | 128.03 | -3.17 | 0.006* | -0.28 (-0.50 to -0.06); *small* |
| KSS (units) | -1.30 | 0.47 | 129.54 | -2.78 | 0.019* | -0.24 (-0.46 to -0.03); *small* |
| Pre-sleep alertness (units) | 0.65 | 0.20 | 128.03 | 3.34 | 0.003* | 0.30 (0.08 to 0.51); *small* |
| **400 mg; 8 h v placebo** | | | | | | |
| Subjective TST (min) | -0.67 | 0.26 | 124.77 | -2.57 | 0.034* | -0.23 (-0.45 to -0.01); *small* |
| Subjective SOL (min) | 11.91 | 5.75 | 126.53 | 2.07 | 0.121 | 0.18 (-0.03 to 0.40); *trivial* |
| Subjective WASO (min) | 5.99 | 6.73 | 126.79 | 0.89 | 1.000 | 0.08 (-0.13 to 0.29); *trivial* |
| Subjective quality (units) | -0.47 | 0.26 | 128.60 | -1.80 | 0.223 | -0.16 (-0.37 to 0.05); *trivial* |
| KSS (units) | -0.73 | 0.47 | 129.54 | -1.57 | 0.358 | -0.14 (-0.35 to 0.07); *trivial* |
| Pre-sleep alertness (units) | 0.25 | 0.20 | 128.55 | 1.25 | 0.64 | 0.11 (-0.10 to 0.32); *trivial* |
| **400 mg; 12 h v placebo** | | | | | | |
| Subjective TST (min) | -0.56 | 0.25 | 124.27 | -2.21 | 0.087 | -0.20 (-0.42 to 0.02); *small* |
| Subjective SOL (min) | 11.60 | 5.61 | 125.57 | 2.07 | 0.122 | 0.19 (-0.03 to 0.40); *trivial* |
| Subjective WASO (min) | 3.10 | 6.64 | 126.44 | 0.47 | 1.000 | 0.04 (-0.17 to 0.25); *trivial* |
| Subjective quality (units) | -0.44 | 0.26 | 128.03 | -1.67 | 0.292 | -0.15 (-0.36 to 0.07); *trivial* |
| KSS (units) | -0.73 | 0.47 | 129.03 | -1.54 | 0.376 | -0.14 (-0.35 to 0.08); *trivial* |
| Pre-sleep alertness (units) | 0.22 | 0.20 | 128.03 | 1.11 | 0.803 | 0.10 (-0.11 to 0.31); *trivial* |
| **100 mg; 4 h v placebo** | | | | | | |
| Subjective TST (min) | -0.28 | 0.26 | 124.48 | -1.10 | 0.823 | -0.10 (-0.31 to 0.12); *trivial* |
| Subjective SOL (min) | 6.74 | 5.68 | 126.04 | 1.19 | 0.712 | 0.11 (-0.11 to 0.32); *trivial* |
| Subjective WASO (min) | -6.28 | 6.73 | 126.79 | -0.93 | 1.000 | -0.08 (-0.30 to 0.13); *trivial* |
| Subjective quality (units) | -0.27 | 0.26 | 128.60 | -1.01 | 0.938 | -0.09 (-0.30 to 0.12); *trivial* |
| KSS (units) | -0.43 | 0.47 | 129.54 | -0.92 | 1.000 | -0.08 (-0.29 to 0.13); *trivial* |
| Pre-sleep alertness (units) | 0.07 | 0.20 | 128.55 | 0.37 | 1.000 | 0.03 (-0.18 to 0.24); *trivial* |
| **100 mg; 8 h v placebo** | | | | | | |
| Subjective TST (min) | -0.13 | 0.26 | 124.22 | -0.49 | 1.000 | -0.04 (-0.26 to 0.17); *trivial* |
| Subjective SOL (min) | 2.46 | 5.74 | 125.50 | 0.43 | 1.000 | 0.04 (-0.18 to 0.25); *trivial* |
| Subjective WASO (min) | -1.61 | 6.80 | 126.37 | -0.24 | 1.000 | -0.02 (-0.23 to 0.19); *trivial* |
| Subjective quality (units) | -0.30 | 0.26 | 128.60 | -1.14 | 0.774 | -0.10 (-0.31 to 0.11); *trivial* |
| KSS (units) | -0.34 | 0.47 | 129.54 | -0.73 | 1.000 | -0.06 (-0.28 to 0.15); *trivial* |
| Pre-sleep alertness (units) | 0.25 | 0.20 | 128.55 | 1.25 | 0.640 | 0.11 (-0.10 to 0.32); *trivial* |
| **100 mg; 12 h v placebo** | | | | | | |
| Subjective TST (min) | -0.58 | 0.26 | 124.48 | -2.28 | 0.073 | -0.20 (-0.42 to 0.01); *small* |
| Subjective SOL (min) | 7.09 | 5.68 | 126.04 | 1.25 | 0.642 | 0.11 (-0.10 to 0.33); *trivial* |
| Subjective WASO (min) | 1.37 | 6.73 | 126.79 | 0.20 | 1.000 | 0.02 (-0.20 to 0.23); *trivial* |
| Subjective quality (units) | -0.26 | 0.26 | 128.60 | -0.98 | 0.982 | -0.09 (-0.30 to 0.13); *trivial* |
| KSS (units) | -0.83 | 0.47 | 130.00 | -1.76 | 0.242 | -0.15 (-0.37 to 0.06); *trivial* |
| Pre-sleep alertness (units) | 0.16 | 0.20 | 128.55 | 0.81 | 1.000 | 0.07 (-0.14 to 0.28); *trivial* |
| **DOSE CONTRASTS** | | | | | | |
| **400 mg; 4 h v 100 mg; 4 h** | | | | | | |
| Subjective TST (min) | -0.98 | 0.26 | 124.48 | -3.82 | 0.001* | -0.34 (-0.56 to -0.12); *small* |
| Subjective SOL (min) | 24.56 | 5.61 | 125.48 | 4.38 | <0.001* | 0.39 (0.17 to 0.61); *small* |
| Subjective WASO (min) | 16.86 | 6.64 | 126.35 | 2.54 | 0.037* | 0.23 (0.01 to 0.44); *small* |
| Subjective quality (units) | -0.56 | 0.26 | 128.60 | -2.12 | 0.107 | -0.19 (-0.40 to 0.03); *trivial* |
| KSS (units) | -0.87 | 0.46 | 129.03 | -1.89 | 0.184 | -0.17 (-0.38 to 0.05); *trivial* |
| Pre-sleep alertness (units) | 0.58 | 0.20 | 128.55 | 2.93 | 0.012* | 0.26 (0.04 to 0.47)*; trivial* |
| **400 mg; 8 h v 100 mg; 8 h** | | | | | | |
| Subjective TST (min) | -0.54 | 0.26 | 125.01 | -2.06 | 0.124 | -0.18 (-0.40 to 0.03); *trivial* |
| Subjective SOL (min) | 9.45 | 5.82 | 127.03 | 1.62 | 0.322 | 0.14 (-0.07 to 0.36); *trivial* |
| Subjective WASO (min) | 7.60 | 6.82 | 127.15 | 1.12 | 0.800 | 0.10 (-0.11 to 0.31); *trivial* |
| Subjective quality (units) | -0.17 | 0.27 | 129.19 | -0.65 | 1.000 | -0.06 (-0.27 to 0.15); *trivial* |
| KSS (units) | -0.39 | 0.46 | 129.03 | -0.85 | 1.000 | -0.08 (-0.29 to 0.14); *trivial* |
| Pre-sleep alertness (units) | 0.00 | 0.20 | 129.10 | 0.00 | 1.000 | 0.00 (0.00 to 0.00); *trivial* |
| **400 mg; 12 h v 100 mg; 12 h** | | | | | | |
| Subjective TST (min) | 0.02 | 0.25 | 124.21 | 0.10 | 1.000 | 0.01 (-0.21 to 0.22); *trivial* |
| Subjective SOL (min) | 4.52 | 5.61 | 125.48 | 0.81 | 1.000 | 0.07 (-0.14 to 0.29); *trivial* |
| Subjective WASO (min) | 1.74 | 6.64 | 126.35 | 0.26 | 1.000 | 0.02 (-0.19 to 0.24); *trivial* |
| Subjective quality (units) | -0.18 | 0.26 | 128.60 | -0.67 | 1.000 | -0.06 (-0.27 to 0.15); *trivial* |
| KSS (units) | 0.11 | 0.47 | 130.00 | 0.22 | 1.000 | 0.02 (-0.19 to 0.23); *trivial* |
| Pre-sleep alertness (units) | 0.06 | 0.20 | 128.55 | 0.30 | 1.000 | 0.03 (-0.19 to 0.24); *trivial* |
| **TIMING CONTRASTS** | | | | | | |
| **400 mg; 4 h v 400 mg; 8h** | | | | | | |
| Subjective TST (min) | -0.59 | 0.26 | 124.22 | -2.28 | 0.073 | -0.21 (-0.42 to 0.01); *small* |
| Subjective SOL (min) | 19.39 | 5.68 | 125.95 | 3.41 | 0.003* | 0.30 (0.09 to 0.52); *small* |
| Subjective WASO (min) | 4.59 | 6.64 | 126.35 | 0.69 | 1.000 | 0.06 (-0.15 to 0.27); *trivial* |
| Subjective quality (units) | -0.35 | 0.26 | 128.60 | -1.34 | 0.550 | -0.12 (-0.33 to 0.09); *trivial* |
| KSS (units) | -0.57 | 0.46 | 129.03 | -1.23 | 0.667 | -0.11 (-0.32 to 0.10); *trivial* |
| Pre-sleep alertness (units) | 0.41 | 0.20 | 128.55 | 2.05 | 0.127 | 0.18 (-0.03 to 0.39); *trivial* |
| **400 mg; 4 h v 400 mg; 12h** | | | | | | |
| Subjective TST (min) | -0.70 | 0.25 | 124.27 | -2.77 | 0.020* | -0.25 (-0.47 to -0.03); *small* |
| Subjective SOL (min) | 19.70 | 5.54 | 125.04 | 3.56 | 0.002* | 0.32 (0.10 to 0.54); *small* |
| Subjective WASO (min) | 7.48 | 6.56 | 126.02 | 1.14 | 0.769 | 0.10 (-0.11 to 0.32); *trivial* |
| Subjective quality (units) | -0.39 | 0.26 | 128.03 | -1.50 | 0.406 | -0.13 (-0.35 to 0.08); *trivial* |
| KSS (units) | -0.57 | 0.47 | 129.54 | -1.22 | 0.673 | -0.11 (-0.32 to 0.10); *trivial* |
| Pre-sleep alertness (units) | -0.09 | 0.20 | 129.10 | -0.43 | 1.000 | -0.04 (-0.25 to 0.17); *trivial* |
| **400 mg; 8 h v 400 mg; 12h** | | | | | | |
| Subjective TST (min) | -0.11 | 0.26 | 124.48 | -0.43 | 1.000 | -0.04 (-0.25 to 0.18); *trivial* |
| Subjective SOL (min) | 0.31 | 5.68 | 125.95 | 0.05 | 1.000 | 0.01 (-0.21 to 0.22); *trivial* |
| Subjective WASO (min) | 2.89 | 6.64 | 126.35 | 0.44 | 1.000 | 0.04 (-0.17 to 0.25); *trivial* |
| Subjective quality (units) | -0.04 | 0.26 | 128.60 | -0.15 | 1.000 | -0.01 (-0.22 to 0.20); *trivial* |
| KSS (units) | 0.00 | 0.47 | 129.54 | -0.01 | 1.000 | 0.00 (-0.21 to 0.21); *trivial* |
| Pre-sleep alertness (units) | 0.03 | 0.20 | 128.55 | 0.15 | 1.000 | 0.01 (-0.20 to 0.22); *trivial* |
| **100 mg; 4 h v 100 mg; 8h** | | | | | | |
| Subjective TST (min) | -0.16 | 0.26 | 124.70 | -0.60 | 1.000 | -0.05 (-0.27 to 0.16); *trivial* |
| Subjective SOL (min) | 4.27 | 5.75 | 126.52 | 0.74 | 1.000 | 0.07 (-0.15 to 0.28); *trivial* |
| Subjective WASO (min) | -4.68 | 6.82 | 127.15 | -0.69 | 1.000 | -0.06 (-0.27 to 0.15); *trivial* |
| Subjective quality (units) | 0.03 | 0.27 | 129.19 | 0.12 | 1.000 | 0.01 (-0.20 to 0.22); *trivial* |
| KSS (units) | -0.09 | 0.46 | 129.03 | -0.19 | 1.000 | -0.02 (-0.23 to 0.19); *trivial* |
| Pre-sleep alertness (units) | -0.17 | 0.20 | 129.10 | -0.87 | 1.000 | -0.08 (-0.29 to 0.14); *trivial* |
| **100 mg; 4 h v 100 mg; 12h** | | | | | | |
| Subjective TST (min) | 0.30 | 0.26 | 124.42 | 1.18 | 0.721 | 0.11 (-0.11 to 0.32); *trivial* |
| Subjective SOL (min) | -0.35 | 5.67 | 125.95 | -0.06 | 1.000 | -0.01 (-0.22 to 0.21); *trivial* |
| Subjective WASO (min) | -7.65 | 6.73 | 126.70 | -1.14 | 0.773 | -0.10 (-0.31 to 0.11); *trivial* |
| Subjective quality (units) | -0.01 | 0.27 | 129.19 | -0.03 | 1.000 | 0.00 (-0.21 to 0.21); *trivial* |
| KSS (units) | 0.41 | 0.47 | 129.46 | 0.87 | 1.000 | 0.08 (-0.14 to 0.29); *trivial* |
| Pre-sleep alertness (units) | 0.44 | 0.20 | 128.03 | 2.23 | 0.083 | 0.20 (-0.02 to 0.41); *small* |
| **100 mg; 8 h v 100 mg; 12h** | | | | | | |
| Subjective TST (min) | 0.46 | 0.26 | 124.70 | 1.76 | 0.242 | 0.16 (-0.06 to 0.37); *trivial* |
| Subjective SOL (min) | -4.62 | 5.75 | 126.52 | -0.80 | 1.000 | -0.07 (-0.28 to 0.14); *trivial* |
| Subjective WASO (min) | -2.97 | 6.82 | 127.15 | -0.44 | 1.000 | -0.04 (-0.25 to 0.17); *trivial* |
| Subjective quality (units) | -0.04 | 0.27 | 129.19 | -0.15 | 1.000 | -0.01 (-0.22 to 0.20); *trivial* |
| KSS (units) | 0.49 | 0.47 | 129.46 | 1.05 | 0.883 | 0.09 (-0.12 to 0.30); *trivial* |
| Pre-sleep alertness (units) | 0.09 | 0.20 | 129.10 | 0.44 | 1.000 | 0.04 (-0.17 to 0.25); *trivial* |
| Abbreviations: CI– confidence interval; df- degrees of freedom; KSS- Karolinska Sleepiness Scale; SOL- sleep onset latency; TST- total sleep time; WASO- wake after sleep onset  Notes: * p < 0.05; 95% CI adjusted for multiple comparisons | | | | | | |
